# Ion Fluxes through K_Ca_2 (SK) and Ca_v_1 (L-type) Channels Contribute to Chronoselectivity of Adenosine A_1_ Receptor-Mediated Actions in Spontaneously Beating Rat Atria

**DOI:** 10.3389/fphar.2016.00045

**Published:** 2016-03-07

**Authors:** Bruno Bragança, Nádia Oliveira-Monteiro, Fátima Ferreirinha, Pedro A. Lima, Miguel Faria, Ana P. Fontes-Sousa, Paulo Correia-de-Sá

**Affiliations:** ^1^Laboratório de Farmacologia e Neurobiologia – Center for Drug Discovery and Innovative Medicines (MedInUP), Instituto de Ciências Biomédicas Abel Salazar (ICBAS), Universidade do Porto (UP)Porto, Portugal; ^2^Departamento de Química e Bioquímica, Faculdade de Ciências, Centro de Química e Bioquímica, Universidade de LisboaLisboa, Portugal

**Keywords:** adenosine, atria, negative chronotropism and inotropism, potassium channels, L-type voltage-sensitive calcium channels

## Abstract

Impulse generation in supraventricular tissue is inhibited by adenosine and acetylcholine via the activation of A_1_ and M_2_ receptors coupled to inwardly rectifying GIRK/K_IR_3.1/3.4 channels, respectively. Unlike M_2_ receptors, bradycardia produced by A_1_ receptors activation predominates over negative inotropy. Such difference suggests that other ion currents may contribute to adenosine chronoselectivity. In isolated spontaneously beating rat atria, blockade of K_Ca_2/SK channels with apamin and Ca_v_1 (L-type) channels with nifedipine or verapamil, sensitized atria to the negative inotropic action of the A_1_ agonist, R-PIA, without affecting the nucleoside negative chronotropy. Patch-clamp experiments in the whole-cell configuration mode demonstrate that adenosine, via A_1_ receptors, activates the inwardly-rectifying GIRK/K_IR_3.1/K_IR_3.4 current resulting in hyperpolarization of atrial cardiomyocytes, which may slow down heart rate. Conversely, the nucleoside inactivates a small conductance Ca^2+^-activated K_Ca_2/SK outward current, which eventually reduces the repolarizing force and thereby prolong action potentials duration and Ca^2+^ influx into cardiomyocytes. Immunolocalization studies showed that differences in A_1_ receptors distribution between the sinoatrial node and surrounding cardiomyocytes do not afford a rationale for adenosine chronoselectivity. Immunolabelling of K_IR_3.1, K_Ca_2.2, K_Ca_2.3, and Ca_v_1 was also observed throughout the right atrium. Functional data indicate that while both A_1_ and M_2_ receptors favor the opening of GIRK/K_IR_3.1/3.4 channels modulating atrial chronotropy, A_1_ receptors may additionally restrain K_Ca_2/SK activation thereby compensating atrial inotropic depression by increasing the time available for Ca^2+^ influx through Ca_v_1 (L-type) channels.

## Introduction

Intravenous bolus of adenosine is clinically useful for prompt conversion of paroxysmal supraventricular tachycardia to sinus rhythm and to control ventricular contraction rate in atrial fibrillation (Savelieva and Camm, 2008; Lim et al., 2009). The use of the nucleoside is preferred mostly due to its rapid onset, short half-life (ranging from 1 to 10 s) and lesser hypotensive effect as compared to other previously recommended drugs, such as the Ca_v_1 (L-type) channel blocker verapamil (Blomström-Lundqvist et al., 2003). Adenosine affects many aspects of cardiac function, including heart rate, contractility and coronary flow through the activation of G protein-coupled A_1_, A_2A_, A_2B_, and A_3_ receptors (Mustafa et al., 2009). The A_1_ receptor promoter is highly active in the atrium as compared to the ventricle, resulting in high A_1_ receptor mRNA levels (Rivkees et al., 1999). This leads to a dominant localization of A_1_ receptors in atrial cardiomyocytes where they exert direct inhibitory effects on chronotropy and dromotropy (Shryock and Belardinelli, 1997; Auchampach and Bolli, 1999), as well as indirect anti-β-adrenergic inotropic responses by opposing the responses of sympathetic nerves activation and β_1_ receptors stimulation (Dobson, 1983; Romano et al., 1991).

Like muscarinic M_2_ receptors which are responsible for the negative chronotropic and inotropic effects of acetylcholine, adenosine A_1_ receptor effects are mediated by hyperpolarization of sinoatrial (SA) node cells as well as cells of the atrioventricular node primarily by inducing outward potassium currents through βγ subunits of G protein-coupled inwardly rectifying K^+^ channels (GIRK or K_IR_3.1∕3.4) (Belardinelli and Isenberg, 1983; Kurachi et al., 1986; Yatani et al., 1987; Mubagwa and Flameng, 2001). Due to different expression levels of A_1_ and M_2_ receptors, the maximal GIRK/K_IR_3.1∕3.4 current that can be activated by endogenous adenosine is smaller than the current triggered by cholinergic agonists (Kurachi et al., 1986). Activation of GIRK/K_IR_3.1/3.4 currents causes a reduction in the action potential duration, thereby decreasing the time available for Ca^2+^ influx through Ca_v_1 (L-type) channels and, hence, heart rate and the force of muscle contraction in atrial myocardium (Urquhart et al., 1993; Belardinelli et al., 1995; Neumann et al., 1999). Negative inotropy may, however, be partially compensated by modulation of multiple downstream targets of G proteins, which may include Ca^2+^ influx through Ca_v_1 (L-type) channels (Wang et al., 2013). The predominant and better-studied Ca_v_1.2 channel isoform coexist with the Ca_v_1.3 in the SA node and atrioventricular node where they participate in pacemaking by different mechanisms (Mangoni and Nargeot, 2008). Despite the extensive literature on this subject, several questions remain unanswered concerning the contribution of distinct K^+^ and Ca^2+^ channels to differential pharmacological responses of atrial cardiomyocytes to both adenosine and acetylcholine.

Interestingly, it has been shown that the negative chronotropic and inotropic responses to adenosine may be differentiated at the postreceptor transduction level in the dog's heart (Oguchi et al., 1995). Supersensitive chronotropic and dromotropic effects have also been described for adenosine during isoproterenol-infusion in the human ventricle *in vivo* after heart transplantation, whereas the nucleoside did not reduce the isoproterenol-induced increase in contractility (Koglin et al., 1996). In this study, we show that the negative chronotropic effect caused by adenosine A_1_ receptors activation is evidenced at much lower concentrations than the negative inotropic action of the nucleoside, which is in clear contrast to the M_2_-receptor-mediated cardiodepression operated by acetylcholine. Given the clinical relevance of this finding and the lack of our knowledge regarding the contribution of K^+^ and Ca^2+^ channel subtypes to adenosine chronoselectivity, we tested the effect of the nucleoside in the absence and in the presence of specific K^+^ and Ca^2+^ channel blockers (see Table 1) in rat atria with intact SA rhythm and in voltage-clamp experiments using acutely dissociated atrial cadiomyocytes. For comparison purposes, we also evaluated whether these channel blockers modulate M_2_ receptors activation, since this is the predominant cholinergic receptor subtype in atrial tissue of most mammalian species (Peralta et al., 1987; Hulme et al., 1990; Wang et al., 2001; Krejci and Tucek, 2002). Additionally, we investigated the regional distribution of the involved receptors (e.g., A_1_ and M_2_) and channels (e.g., Ca^2+^ and K^+^) in the right atrium and SA node by immunofluorescence confocal microscopy.

**Table 1:** **List of used drugs and their pharmacological characteristics**.

**Drug**	**Target selectivity**	**Concentration range**	**Supplier**
Adenosine	Endogenous adenosine receptor ligand	0.001–3 mM	Sigma-Aldrich
R-PIA	Selective A_1_ receptor agonist	0.001–1 μM	Sigma-Aldrich
CGS21680C	Selective A_2A_ receptor agonist	0.003–1 μM	Sigma-Aldrich
DPCPX	Selective A_1_ receptor antagonist^a^	100 nM	Sigma Aldrich
Oxotremorine	Preferential M_2_ receptor agonist (≥98%)^b^	0.003–3 μM	Tocris Cookson Inc.
AF-DX 116	Preferential muscarinic M_2_ receptor antagonist^c^	10 μM	Tocris Cookson Inc.
4-AP	Nonspecific voltage-dependent K_v_ channel blocker^d^	10 μM	Sigma-Aldrich
Glibenclamide	Selective blocker of ATP-sensitive inward rectifier K_ATP_/K_IR_6 channels	10 μM	Ascent Scientific
Tertiapin Q	Potent blocker of inward rectifier GIRK/K_IR_ currents with high affinity for K_IR_ 3.1/3.4 channels^e^	300 nM	Ascent Scientific
Apamin	Selective blocker of small-conductance Ca^2+^-activated K_Ca_2/SK channels^f^	30 nM or 0.003–1 μM	Sigma-Aldrich
Nifedipine	Selective blocker of high-voltage Ca_v_1 (L-type) channels	1 μM	Sigma-Aldrich
Verapamil	Selective blocker of high-voltage Ca_v_1 (L-type) channels	1 μM or 0.03–10 μM	Tocris Cookson Inc.
Mibefradil	Moderately selective blocker of high-voltage Ca_v_3 (T-type) channels. Displays IC_50_ values of 2.7 μM and 18.6 μM for T-type and L-type channels, respectively	3 μM	Tocris Cookson Inc.

a *Used as a selective high-affinity antagonist for adenosine A_1_ receptors (K_i_~0.45 nM) with more than 700-fold selectivity over other adenosine receptors, namely the A_2A_ receptor (Lohse et al., 1987)*.

b *Cited as the predominant form of muscarinic receptors present in the heart of various mammalian species, including humans (Peralta et al., 1987; Hulme et al., 1990; Caulfield, 1993)*.

c *Used as a preferential M_2_ (and M_4_) receptor antagonist (pK_B_ 7.1–7.2), the most expressed receptor in the heart (Caulfield, 1993)*.

d *Used as voltage-dependent K_v_ channel blocker (e.g., Bardou et al., 2001)*.

e *Tertiapin Q is a high affinity and potent blocker of inward-rectifier K^+^ currents that is widely used for inhibiting GIRK1/4 (K_IR_3.1/3.4) channels in the nanomolar concentration range (see e.g., Jin and Lu, 1998; Whorton and MacKinnon, 2011)*.

f *Prototypical potent and highly selective inhibitor of the small-conductance Ca^2+^-activated K^+^-channel (K_Ca_2, SK); complete blockade of K_Ca_2 (SK) currents were observed at 100 nM apamin (Hugues et al., 1982)*.

## Materials and methods

### Animals

Wistar rats (*Rattus norvegicus*; 250–300 g) of either sex (Charles River—CRIFFA, Barcelona, Spain; Vivarium ICBAS, Porto, Portugal) were housed in a temperature-controlled (21°C) room with a regular 12:12-h light-dark cycle. The animals were provided free access to standard laboratory chow and water. Animal care and experimental procedures were carried out in accordance with the UK Animals (Scientific Procedures) Act 1986 and followed the European Communities Council Directive of 24 November 1986 (86/609/EEC) and the National Institutes of Health Guide for Care and Use of Laboratory animals (NIH Publications No. 80–23) revised 1996. All studies involving animals are reported in accordance with ARRIVE guidelines for reporting experiments involving animals (McGrath et al., 2010).

### Isolated perfused spontaneously beating rat atria

Isolated perfused beating atria were prepared using a previously described method (Kitazawa et al., 2009), with some modifications. In brief, hearts were rapidly excised after decapitation followed by exsanguination (Rodent guillotine, Stoelting 51330), and placed in a physiological solution (Tyrode's solution) composed of (mM): NaCl 137; KCl 2.7; CaCl_2_ 1.8; MgCl_2_1; NaH_2_PO_4_ 0.4; NaHCO_3_ 11.9; glucose 11.2 and gassed with 95% O_2_ + 5% CO_2_ (at pH 7.4). In some of the experiments the concentration of KCl was raised from 2.7 (see e.g., De Biasi et al., 1989) to 4.7 mM. Hearts were allowed to beat freely for a few seconds at room temperature, to empty its blood content. The paired rat atria with the SA node region were dissected out, cleaned of fatty tissues, and suspended in a 14-mL organ bath containing gassed Tyrode's solution at 37°C. Each auricular appendage was tied and connected with thread to the organ bath wall and to an isometric force transducer (MLT050/D; AD Instruments, Colorado Springs, CO, USA). Changes in isometric tension were recorded continuously using a PowerLab data acquisition system (Chart 5, version 4.2; AD Instruments, Colorado Springs, CO, USA). The preparations were allowed to equilibrate for 30–40 min. During this time, the preparations were superfused with Tyrode's solution and the tension was adjusted to 9.8 mN. This procedure allows atria (with intact SA node) to progressively recover rhythmic spontaneous beatings; preparations with spontaneous atrial rate below 200 beats min^−1^ or exhibiting rhythm variations above 10 beats min^−1^ during equilibrium were discarded to ensure measurements were made in atria with intact primary pacemaker SA node activity. None of the preparations exhibited noticeable signs of ectopic-activity caused by secondary pacemakers, usually related to asynchronous and abnormal contractions. Under these experimental conditions, spontaneously beating rat atria respond to muscarinic and β-adrenergic stimulation, but are unaffected by the application of atropine or propranolol alone used in concentrations high enough (10 μM) to prevent the effects of acetylcholine (100 μM) and isoproterenol (30 nM), respectively. Thus, myographic recordings reported in this study include rate (chronotropic effect) and contractile force (inotropic effect) of spontaneously beating atria measured in the absence of cholinergic and/or adrenergic tone.

In some of the experiments, isometric tension was tested at a fixed frequency of 240 beats min^−1^ commanded by electric field stimulation of the preparations as an index of inotropy measured without being affected by concurrent changes in chronotropy. Electric atrial pacing (4 Hz, 100 V, 0.5 ms) was performed using a Grass S48 Stimulator (Quincy, MA, USA) via two platinum electrodes positioned on each side of the preparations.

### Experimental design

After reaching a steady-state (Control values shown in Table 2), the perfusion with Tyrode's solution was stopped and the preparations were incubated with increasing concentrations of **R**-(-)-N^6^-(2-phenylisopropyl)adenosine (R-PIA, 0.001–1 μM), a stable adenosine A_1_ receptor agonist, or oxotremorine (0.003–3 μM), a muscarinic M_2_ receptor agonist, either in the absence or in the presence of 1,3-dipropyl-8-cyclopentylxanthine (DPCPX, 100 nM) or AF-DX 116 (10 μM), which block A_1_ and M_2_ receptors, respectively. Adenosine (0.001–3 mM) and the adenosine A_2A_ receptor agonist, 2-p-(2-carboxyethyl)phenethylamino-5′-N-ethylcarboxamidoadenosine (CGS 21680C, 0.003–1 μM), were also tested in some of the experiments. Agonists were added cumulatively into the bathing solution at 2 min intervals, as this time was considered sufficient for each concentration to equilibrate with the preparation and to cause a maximal response under the present experimental conditions. To examine the role of K^+^ and Ca^2+^ channel currents in the effects of adenosine A_1_ and muscarinic M_2_ receptors activation, concentration-response curves to R-PIA (0.001–1 μM) and oxotremorine (0.003–3 μM) were established in the presence of the following inhibitors: 4-aminopyridine (4-AP, 10 μM), a nonspecific voltage-dependent K_v_ channel blocker, glibenclamide (10 μM), a selective blocker of ATP-sensitive K_ATP_/K_IR_6 channels, tertiapin Q (300 nM), a blocker of GIRK/K_IR_ channels with high affinity for K_IR_3.1/3.4 channels, apamin (30 nM), an inhibitor of small-conductance Ca^2+^-activated K^+^ (K_Ca_2/SK) channels, nifedipine and verapamil (1 μM), selective blockers of high voltage-activated Ca_v_1 (L-type) channels, and mibefradil (3 μM), a low voltage-activated Ca_v_3 (T-type) channel blocker (see Table 1). For the sake of data normalization, all the inhibitors were allowed to equilibrate with the preparations at least for 15 min before application of R-PIA or oxotremorine; values for atrial rate (chronotropic effect) and contractile force (inotropic effect) after reaching the equilibrium with the receptor antagonists or ion channel inhibitors are shown in Table 2. The concentrations of the inhibitors in this study were within the range of channel selectivity described in the literature. Since, blockade of K^+^ channels can stimulate Ca^2+^ influx through voltage-sensitive Ca_v_1 (L-type) channels, we examined the responses of the rat spontaneously beating atria to cumulative application of verapamil (0.03–10 μM) in the absence and presence of apamin (30 nM) or tertiapin Q (300 nM). In another group of experiments, the myographic effects of increasing concentrations of apamin (0.003–1 μM) in the absence and presence of verapamil (1 μM) were also recorded. The protocol for drug application was identical to that described for the studies using R-PIA and oxotremorine (see **Figure 2A**).

**Table 2:** **Influence of receptor antagonists and ion channel inhibitors on chronotropism and inotropism of spontaneously beating rat atria**.

**Protocols**	**Chronotropism (*beats.min*^1^)**	**Inotropism (*mN*)**	***n***
**R-PIA SERIES**			
**Control**	**200 ± 5**	**3.9 ± 0.7**	**40**
+ DPCPX (100 nM)	220 ± 4	3.8 ± 0.2	4
+ 4-AP (10 μM)	210 ± 15	5.1 ± 0.4^*^	6
+ Glibenclamide (10 μM)	214 ± 26	4.8 ± 0.6^*^	5
+ Tertiapin Q (300 nM)	206 ± 14	3.7 ± 0.3	5
+ Apamin (30 nM)	221 ± 11	4.5 ± 0.5	8
+ Nifedipine (1 μM)	148 ± 26^*^	3.7 ± 1.1	5
+ Verapamil (1 μM)	160 ± 17^*^	3.8 ± 0.6	5
+ Mibefradil (3 μM)	169 ± 16^*^	3.5 ± 0.2	7
**OXOTREMORINE SERIES**			
**Control**	**213 ± 5**	**3.5 ± 0.3**	**17**
+ AF-DX 116 (10 μM)	225 ± 13	3.2 ± 0.1	6
+ 4-AP (10 μM)	209 ± 21	4.6 ± 0.4^*^	5
+ Glibenclamide (10 μM)	220 ± 26	4.1 ± 0.4^*^	6
+ Tertiapin Q (300 nM)	228 ± 15	3.6 ± 0.5	5
+ Apamin (30 nM)	220 ± 13	4.0 ± 0.5	7
+ Nifedipine (1 μM)	142 ± 26^*^	3.6 ± 0.5	5
+ Verapamil (1 μM)	165 ± 17^*^	3.4 ± 0.4	6
+ Mibefradil (3 μM)	164 ± 15^*^	3.3 ± 0.2	6
**VERAPAMIL SERIES**			
**Control**	**205 ± 11**	**3.3 ± 0.3**	**14**
+ Apamin (30 nM)	210 ± 19	3.9 ± 0.4	7
+ Tertiapin Q (300 nM)	202 ± 11	3.4 ± 0.2	5
**APAMIN SERIES**			
**Control**	**218 ± 6**	**3.4 ± 0.3**	**9**
+ Verapamil (1 μM)	157 ± 20^*^	3.5 ± 0.8	7

*Receptor antagonists and ion channel inhibitors were allowed to equilibrate with the preparations during 15 min before R-PIA (0.001–1 μM), oxotremorine (0.003–3 μM), verapamil (0.03–10 μM), or apamin (0.003–1 μM) application. Shown are values for atrial rate (chronotropic effect) and contractile force (inotropic effect) in the absence (Control or vehicle) and in presence of receptor antagonists and ion channel inhibitors after reaching the equilibrium, i.e., measured immediately before incubation with R-PIA, oxotremorine, verapamil, and apamin. The data are expressed as mean ± SEM from an n number of individual experiments (animals)*.

* *P < 0.05 compared with the control situation before incubation with receptor antagonists or ion channel inhibitors*.

### Isolation of atrial cardiomyocytes

Atrial cardiomyocytes were obtained by enzymatic digestion using a Langendorff perfusion apparatus. Rat hearts were quickly removed and washed in HEPES buffer solution (composition in mM: NaCl 135, KCl 5, MgSO_4_ 1.5, NaH_2_PO_4_ 0.33, HEPES 10, CaCl_2_ 0.5, D-glucose 15, adjusted to pH 7.35 with NaOH) containing heparin (50 UI/mL) at room temperature (~20°C). The excised hearts were then catheterized through the aorta and superfused retrogradely at a flow rate of 7 ml.min^−1^ with a nominally calcium-free HEPES buffer solution gassed with 100% O_2_ at 37°C. Five minutes after initiating heart perfusion, the superfusion fluid was supplemented with collagenase II (Worthington Biochemical Corp., 148 U/ml) and protease XIV (Sigma-Aldrich, 10 U/mL) and the calcium concentration was raised to 0.2 mM. As soon as the heart became soft to the touch, the superfusion was stopped (for about 15 min) and atria were dissected free from the ventricles. Isolated atria were then gently minced into small pieces with microdissecting scissors and further digested using a plastic transfer pipette to release single myocytes. To separate single myocytes from non-digested tissue, the cellular suspension was filtered through a 500 μm mesh. The cellular suspension was centrifuged 3 times at 18 g for 3 min. The resulting pellet of each centrifugation was then re-suspended in fresh physiological solution (described above) containing 10 mM 2,3-butanedione monoxime and increasing concentrations of CaCl_2_ to steeply raise the extracellular calcium to a final concentration of 1 mM. This isolation procedure yields ~60–70% of Ca^2+^ tolerant atrial cadiomyocytes with clear myofibrilar striations. Acutely dissociated atrial cardiomyocytes were kept at room temperature and used up to 6 h after their isolation.

### Patch-clamp experiments in isolated atrial cardiomyocytes

Acutely dissociated atrial cardiomyocytes were placed onto 35 mm plastic petri dishes (Nunclon™Δ surface; Nunc, Roskilde, Denmark), which were used as recording chambers mounted on the stage of an inverted microscope. Myocytes were allowed to adhere to the bottom of chamber for 10 min. A gravity-fed system was used for the exchange of extracellular solution (2–3 ml.min^−1^), which had the following composition (in mM): NaCl 135, KCl 5, MgSO_4_ 1.5, NaH_2_PO_4_ 0.33, HEPES 10, CaCl_2_ 1, D-glucose 15, adjusted to pH 7.35 with 1 mM NaOH. The bathing solution was bubbled with 100% O_2_ at room temperature. Atrial cardiomyocytes were voltage-clamped using the whole cell patch-clamp configuration, as described previously (Vicente et al., 2010). Briefly, the patch pipettes (2.80 ± 0.09 MΩ, *n* = 19) were pulled from borosilicate glass capillaries (Science Products GmbH, GB150T-8P) and filled with an internal solution containing (in mM): potassium gluconate 135, KCl 5, NaCl 5, Na_1∕2_HEPES 10, MgCl_2_ 1, EGTA 0.1, Na_2_ATP 2, NaGTP 0.4 (pH 7.3 adjusted with 1 mM KOH; 305 ± 5 mOsm). Only rod-shaped myocytes with no spontaneous contractions at rest were used for experiments. The estimated junction potential for the filling and bathing solution combinations mentioned above is −8.9 mV (calculated with JPCalc 2.00, School of Physiology and Pharmacology University of New South Wales). Data were not corrected for the junction potential. Currents were recorded with an Axopatch 200B electrometer (Axon Instruments Inc., USA) and stored on a PC using the pClamp 6.0.3 software (Axon Instruments Inc., USA) and an analog digital interface (Digidata 1200; Axon Instruments, USA). Signals were acquired at a sampling rate of 5 kHz and filtered at 2 kHz (−3 dB, four pole Bessel). Quantification of currents were made by measuring the peak current 30 ms after the initial voltage step of the command pulse, which accounts for an approximate measure of the peak current but, away enough from the occurrence of the fast *I*_*Na*_, avoiding the contamination of the sodium currents. Current amplitudes were measured in respect to “zero current,” which was given by the current value at −40 mV, a potential where the “expected” physiological current should be zero. Whole-cell capacitance (132 ± 6 pF, *n* = 9) was calculated from the area under the curve fitted to the transient capacitive current produced by 5 mV test depolarizing step from a holding potential of −70 mV. Cells with significant leak currents were rejected. Also, series resistance (5.5 ± 0.3 MΩ, *n* = 14) were monitored throughout the experiments and only recordings with variation < 10% were considered valid.

The holding potential (V_H_) was kept at −70 mV, unless otherwise specified. The Ca^2+^ dependence of outward K^+^ currents was assessed using a two-pulse protocol. A prepulse lasting 50 ms to −10 mV was delivered to trigger Ca^2+^ influx through voltage-activated channels. The prepulse was immediately followed by a second depolarizing pulse to +40 mV for 750 ms to elicit the outward K^+^ current. Before evaluating the voltage-dependence of activation, the current stability was monitored with a set of two-pulse protocols applied every 2 min. Drugs were diluted previously and then included in the superfusion fluid.

### Immunofluorescence staining and confocal microscopy studies

**Figure 1:**
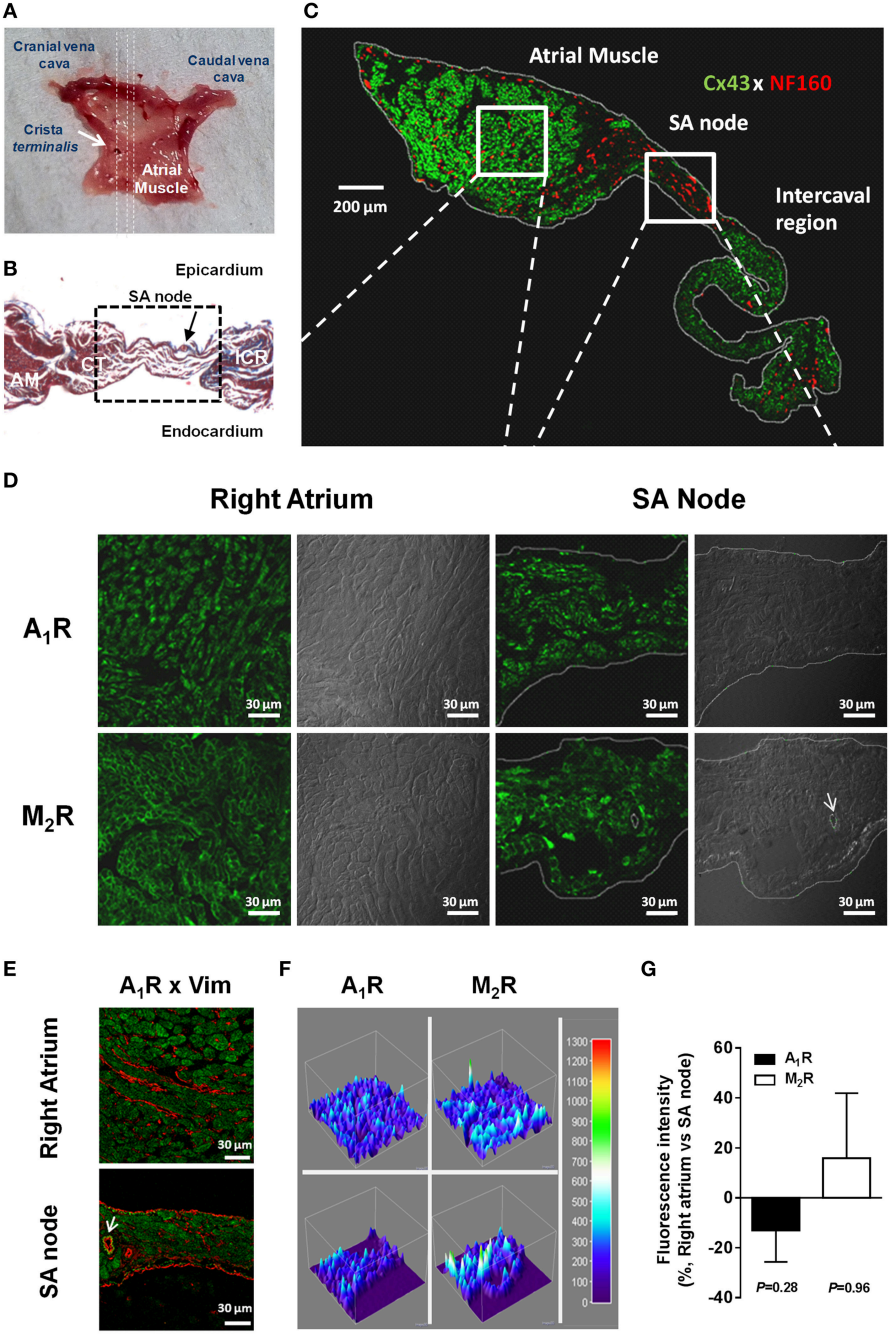
**Anatomical and molecular identification of the rat SA node (A–C). (A)** Endocardial view of a typical rat atrial muscle-SA node preparation. Dashed lines indicate the orientation of cuts performed on the right atrial appendage for Masson's trichrome staining and immunofluorescence analysis. **(B)** High-magnification (400x) images of Masson's trichrome staining of SA node (dashed box) and surrounding regions; AM, atrial muscle; CT, *crista terminalis*; ICR, intercaval region. Red staining, myocytes; blue staining, connective tissue. Black arrow indicates the SA node artery. **(C)** Montage of confocal optical sections showing immunolabelling for connexin 43 protein (Cx43, green signal) and neurofilament 160 (NF160, red signal) throughout the right atrial appendage from the rat. Boxes depict representative areas of the right atrium muscle and SA node used for immunolocalization of receptors and ion channels. **(D)** Representative confocal micrographs of the rat SA node and neighboring atrial muscle showing immunofluorescence labeling of A_1_ (upper panels) and M_2_ (lower panels) receptors; the corresponding differential interference contrast (DIC) images are also shown for comparison. **(E)** Please note that immunolabelling of A_1_ receptors (green) did not co-localize with vimentin (Vim) staining (red), which was used as a fibroblast-cell marker. Similar results were obtained in six additional experiments. The white arrow indicates the SA node artery. **(F)** Tridimensional surface modeling representing immunoreactivity of images depicted in panel **(D)**. Color bar represents relative fluorescence intensity map. **(G)** Graph depicting semi-quantitative analysis of A_1_R and M_2_R expression in right atria and SA node; the ordinates are immunofluorescence intensity ratio between A_1_R and M_2_R staining in paired samples from the right atrium and SA node keeping the image acquisition settings constant. Positive and negative values indicate staining predominance in contractile myocardium and SA node of the right atrium, respectively. Values are mean ± SEM; at least 3 microscopic fields were analyzed per section of the right atrium and SA node obtained from three to seven rats. *P* > 0.05 indicates that no significant differences were found in the expression of A_1_R and M_2_R between the two atrial regions.

Rat hearts were excised (see above) and placed in oxygenated Tyrode's solution at room temperature. Following heart excision the right atrium containing the SA node region and surrounding atrial muscle was accurately isolated. Tissue fragments were stretched to all directions, pinned flat onto cork slices and embedded in Shandon cryomatrix (Thermo Scientific) before frozen in a liquid nitrogen-isopentane bath. Frozen sections with 8 μm thickness were cut perpendicular to the *crista terminalis* of the right atrium (see Figure 1A). Once defrosted, tissue sections were fixed in phosphate buffered saline (PBS) containing 50% acetone and 2% paraformaldehyde. Following fixation, the preparations were washed three times for 10 min each using 0.1 M PBS and incubated with a blocking buffer, consisting in fetal bovine serum 10%, bovine serum albumin 1%, Triton X-100 0.3% in PBS, for 2 h. After blocking and permeabilization, samples were incubated with selected primary antibodies (Table 3) diluted in incubation buffer (fetal bovine serum 5%, serum albumin 1%, Triton X-100 0.3% in PBS), overnight at 4°C. For double immunostaining, antibodies were combined before application to tissue samples. Following the washout of primary antibodies with PBS (3 cycles of 10 min) tissue samples were incubated with species-specific secondary antibodies (Table 3) in the dark for 2 h, at room temperature. Negative controls were carried out by replacing the primary antibodies with non-immune serum; cross-reactivity of the secondary antibodies was tested in control experiments in which primary antibodies were omitted. Finally, tissue samples were mounted on optical-quality glass slides using VectaShield as antifade mounting media (H-1200; Vector Labs) and stored in the dark at 4°C. Observations were performed and analyzed with a laser-scanning confocal microscope (Olympus FluoView, FV1000, Tokyo, Japan). The SA node region is found in close proximity to subepicardial sinus node artery (arrow in Figure 1B). SA node is characterized by a large number of neurofilament 160 (NF-160) positive neuronal fibers (Dobrzynski et al., 2005) and small-size cardiomyocytes that are negative against connexin-43 (Cx43) staining, a gap junction protein ubiquitously expressed in the heart apart from in nodal tissue (van Kempen et al., 1991) (Figure 1C). Cells within the SA node are surrounded by dense connective tissue; collagen fibers (light blue staining) can be differentiated from cardiomyocytes (red staining) with the Masson's trichrome staining (Figure 1B). Vimentin was used as a fibroblast-cell marker in immunofluorescence confocal microscopy studies (see Figure 1E). Semi-quantification of immunofluorescence signals was performed using the FluoView software; at least three images per section of the right atrium and the SA node obtained with a 600X magnification (640 × 640 pixels resolution) were processed. The images were stored in TIFF format with the same resolution and, subsequently, analyzed in 3D Objects Counter plugin for ImageJ® software version 1.50d (National Institutes of Health). This plugin allowed us to measure the integrated pixel density of automatically generated regions of interest (ROI). The average integrated pixel density of all generated ROIs of each image was used to estimate brightness intensity of the specific immunolabelling. Fluorescence intensity ratio for each pair of sections was used as a semi-quantitative approach to evaluate the relative expression of receptors and ionic channels in the right atrium and the SA node. Of note, the acquisition settings on the confocal microscope were kept constant in all optical sections of the right atrium and the SA node from the same animal. Representative images of immunofluorescence staining were used to create tridimensional models representing the immunereactivity intensity by means of the Interactive 3D surface plot v2.33 plugin for ImageJ®. Plugin settings were kept constant in all tridimensional assemblies. The peak height and color represent the magnitude of immunoreactivity intensity. One researcher conducted all semi-quantitative image analysis blindly.

**Table 3:** **Primary and secondary antibodies used in immunohistochemistry experiments**.

**Antigen**	**Code**	**Host**	**Dilution**	**Supplier**
**PRIMARY ANTIBODIES**				
Connexin 43 (Cx43)	ab11370	Rabbit (rb)	1:700	Abcam
Neurofilament 160 (NF-160)	ab7794	Mouse (ms)	1:1000	Abcam
Vimentin	M0725	Mouse (ms)	1:150	Dako
Adenosine receptor A_1_	AB 1587P	Rabbit (rb)	1:100	Chemicon
Muscarinic receptor M_2_	AMR-002	Rabbit (rb)	1:200	Alomone
Small-conductance Ca^2+^-activated K^+^ channel (K_Ca_2.2)	AP10032PU-N	Goat (gt)	1:400	Acris antibodies
Small conductance Ca^2+^-activated K^+^ channels (K_Ca_2.3)	ab83737	Rabbit (rb)	1:300	Abcam
High voltage-activated Ca^2+^ (L-type) channels (Ca_v_α_2_δ−1)	ab2864	Mouse (ms)	1:50	Abcam
G protein-coupled inwardly rectifying K^+^ channels (K_IR_3.1)	ab61191	Rabbit (rb)	1:500	Abcam
**SECONDARY ANTIBODIES**				
Alexa Fluor 488 anti-rb	A-21206	Donkey	1:1500	Molecular probes
Alexa Fluor 568 anti-ms	A-10037	Donkey	1:1500	Molecular probes
Alexa Fluor 568 anti-gt	A-11057	Donkey	1:1500	Molecular probes

### Solutions and chemicals

N-[4-[[1-[2-(6-methyl-2-pyridinyl)ethyl]-4-piperidinyl]carbonyl]phenyl]methanesulfonamide dihydrochloride (E4031) from Tocris Cookson Inc. (Bristol, UK). Adenosine, 4-aminopyridine (4-AP), apamin, 2,3-butanedione monoxime, 2-p-(2-carboxyethyl) phenethylamino-5′-N-ethylcarboxamidoadenosine (CGS21680C), 1,3-dipropyl-8-cyclopentyl-xanthine (DPCPX), nifedipine, propranolol, protease XIV, **R**-(-)-*N*^6^-(2-phenylisopropyl) adenosine (R-PIA) were from Sigma-Aldrich (St. Louis, MO, USA); AF-DX 116, oxotremorine sesquifumarate, mibefradil and verapamil were from Tocris Cookson Inc. (Bristol, UK); glibenclamide and tertiapin Q were from Ascent Scientific (Bristol, UK); Dimethylsulfoxide (DMSO), serum albumin and Triton X-100 were from Merck (Darmstadt, Germany); collagenase II was from Worthington Biochemical Corp (Lakewood, NJ, USA). AF-DX 116 and glibenclamide were made up in DMSO stock solution. DPCPX was made up in 99% DMSO/1% NaOH 1 mmol L^−1^ (v/v). R-PIA, verapamil and nifedipine were made up in ethanol; these solutions were kept protected from the light to prevent photodecomposition. Other drugs were prepared in distilled water. All stock solutions were stored as frozen aliquots at −20°C. Dilutions of these stock solutions were made daily and appropriate solvent controls were done. No statistically significant differences between control experiments, made in the absence or in the presence of the solvents at the maximal concentrations used (0.5% v/v), were observed. The pH of the superfusion solution did not change by the addition of the drugs in the maximum concentrations applied to the preparations.

### Presentation of data and statistical analysis

The isometric contractions were recorded and analyzed before and after the addition of each drug at the desired concentration. Results were presented as percentages of variation compared to baseline, obtained before the administration of the evaluated drug. Concentration-response curves were fitted by non-linear regression using GraphPad Prism 5.04 software (La Jolla, CA, USA) function: log(inhibitor) vs. response. We assumed that both data share best-fit values for top and bottom and a Hill slope equal to 1; in the case of drugs (e.g., R-PIA) showing an intermediate increase in inotropy, we considered that data share best-fit values for bottom (constrained to −100%) in order to estimate pIC_50_ values. Fitting used the least squares method. The data are expressed as mean ± SEM, with *n* indicating the number of animals used for a particular group of experiments. With the exception of patch-clamp experiments using acutely dissociated atrial cardiomyocytes, each rat was used to test only one pair of drug and antagonist/inhibitor. Comparison between concentration-response curves obtained in the absence and in the presence of a receptor antagonist or ion channel inhibitor was performed using two-way ANOVA followed by the Sidak's multiple comparison test. Individual pairs of data were compared using paired Student's *t*-test when parametric data was considered. The means of unmatched groups were compared using unpaired Student's *t*-test with Welch's correction when parametric data was considered. For multiple comparisons, one-way ANOVA followed by Dunnett's modified *t*-test was used. *P* < 0.05 (two-tailed) values were considered to show significant differences between means.

## Results

### Adenosine acting via A_1_ receptors is a chronoselective atrial depressant as compared to the muscarinic M_2_ receptor agonist, oxotremorine

**Figure 2:**
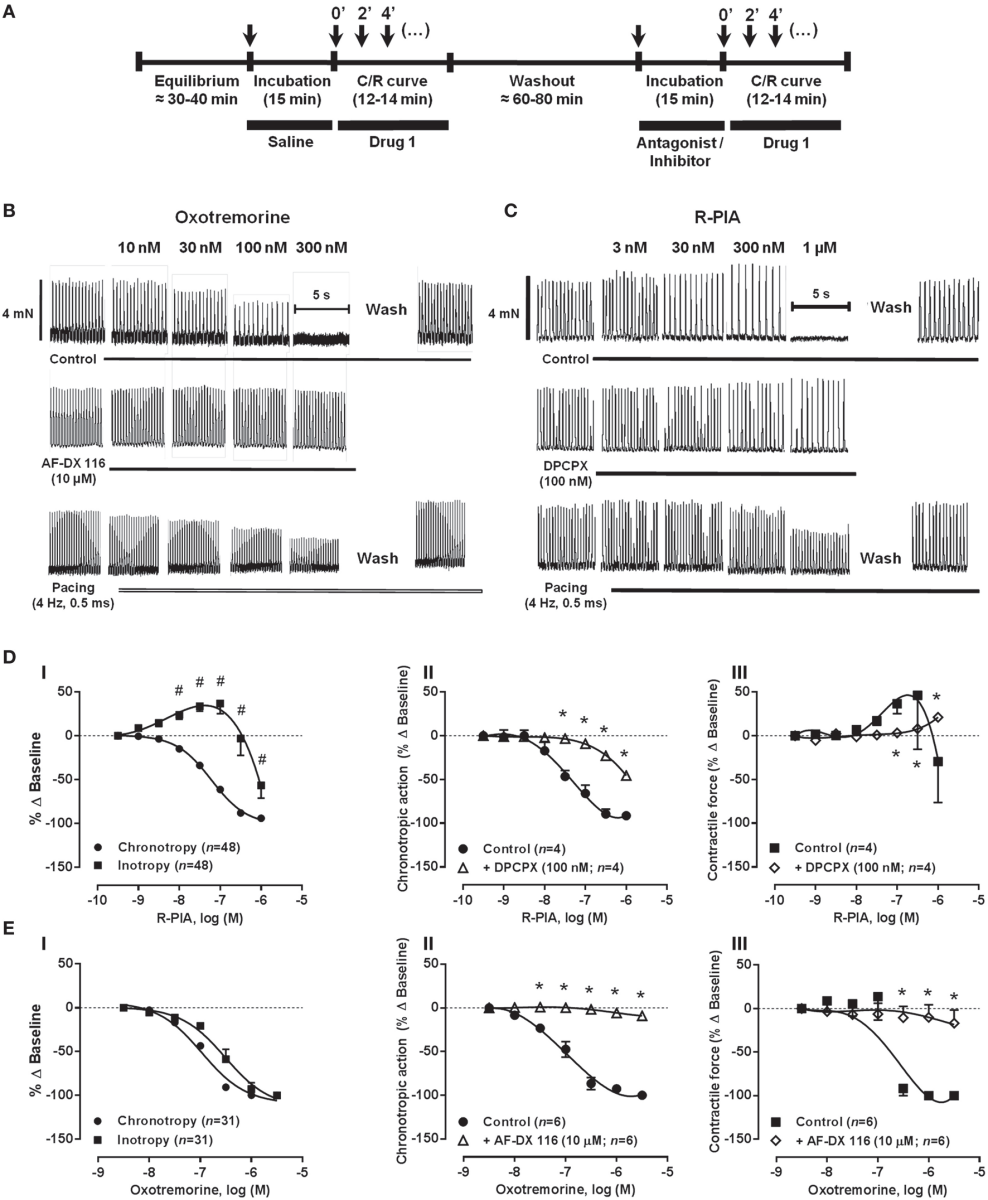
**Schematic representation of the protocol used for drug applications (A)**. Concentration-response C/R curves of oxotremorine **(B,E)** and R-PIA **(C,D)** on the spontaneously beating rat atria in the absence (Control) and in the presence of selective M_2_ (AF-DX 116) and A_1_ (DPCPX) receptor antagonists. The effects of oxotremorine and R-PIA in rat atria electrically-paced at a constant frequency of 240 beats per min (4 Hz) are also shown for comparison (spontaneous atrial rate in control conditions was 218 ± 4 beats.min^−1^, *n* = 10). Oxotremorine (0.003–3 μM) and R-PIA (0.001–1 μM) were applied once every 2 min at increasing concentrations; AF-DX 116 (10 μM) and DPCPX (100 nM) were added to the incubation fluid 15 min before application of oxotremorine or R-PIA. The ordinates are percentage of variation of spontaneous contraction rate (chronotropic effect, **ii**) and mechanical tension (inotropic effect, **iii**) compared to baseline values obtained before application of the corresponding agonist. The data are expressed as mean ± SEM from an *n* number of individual experiments. ^#^*P* < 0.05 compared with agonist-induced percentage of baseline variation on chronotropy; ^*^P < 0.05 compared with the effect of oxotremorine or R-PIA in the absence of receptor antagonists AF-DX 116 and DPCPX, respectively.

Figure 2 shows that activation of adenosine A_1_ and muscarinic M_2_ receptors with R-PIA (0.001–1 μM) and oxotremorine (0.003–3 μM), respectively, decreased in a concentration-dependent manner the rate (negative chronotropic effect) and the force (negative inotropic effect) of spontaneous contractions of rat atria. The negative chronotropic effect of R-PIA (pIC_50_ 7.26 ± 0.04) was evidenced at much lower (*P* < 0.01) concentrations than its negative inotropic action (pIC_50_ 6.14 ± 0.07) (Figure 2Di), whereas oxotremorine-induced depression of both rate and tension of spontaneously beating atria was observed in the same concentration range (pIC_50_ 6.97 ± 0.03 and 6.81 ± 0.10, respectively; *P* > 0.05) (Figure 2Ei). Adenosine (0.001–3 mM) mimicked the negative chronotropic and inotropic effects of the full A_1_ agonist, R-PIA (0.001–1 μM), but the effect of the natural nucleoside was 4 log units less potent than R-PIA (Supplementary Figure 1). Reduction of atrial rate produced by adenosine (pIC_50_ 3.68 ± 0.09) was preferential compared to its ability to decrease tension of spontaneously beating atria (pIC_50_ 2.60 ± 0.16, *n* = 9, *P* < 0.02 vs. negative chronotropy). Unlike adenosine, R-PIA transiently increased (*P* < 0.05) atrial contractile force when applied in 0.03 and 0.1 μM concentrations (Figure 2Di). Likewise, the selective adenosine A_2A_ receptor agonist, CGS21680C, produced a mild positive inotropic effect (maximal increase, ~18% at 300 nM) on spontaneously beating rat atria, when this compound was applied in a similar concentration range (0.003–1 μM) as R-PIA (*n* = 5, data not shown) (see also Dobson and Fenton, 1997). These findings suggest that the A_2A_ receptor has limited importance in the response to adenosine in the isolated spontaneously beating rat atria, yet it may contribute to increase atrial contractile force when significant negative chronotropism concurrently occurs as observed with R-PIA (see Figure 2Di).

Figures 2Dii,iii show that selective blockade of adenosine A_1_ receptors with DPCPX (100 nM), significantly shifted to the right the concentration-response curves for R-PIA (0.001–1 μM) in the spontaneously beating rat atria. The blocking effect of DPCPX was dependent on the concentration (2.5, 10, and 100 nM, *n* = 4−6), thus indicating that the A_1_ receptor must be the dominant receptor involved in the negative chronotropic and inotropic actions of R-PIA. Likewise, data from Figures 2Eii,iii show that depression of rate and tension of spontaneous atrial contractions caused by oxotremorine (0.003–3 μM) were both completely prevented by pre-incubation with the muscarinic M_2_ receptor antagonist, AF-DX 116 (10 μM). Immunolocalization studies showed that A_1_ and M_2_ receptors are evenly expressed in SA node and atrial cardiomyocytes (Figures 1D,F,G), thus indicating that differences in the receptors regional distribution do not account for adenosine chronoselectivity in spontaneously beating rat atria.

A yet unknown postreceptor transduction mechanism to explain the negative chronotropic supersensitivity to adenosine in the dog heart has been hypothesized (Oguchi et al., 1995). Supplementary Figure 2, shows that adenosine negative chronoselectivity might depend on potassium ionic currents since it was significantly (*P* < 0.05) reduced by raising the extracellular concentration of KCl from 2.7 to 4.7 mM (see e.g., De Biasi et al., 1989). Under these conditions, the negative inotropic effect of R-PIA (Supplementary Figure 2D) became evident at the same concentration range (0.003–1 μM) as that needed to slow down atrial rate (Supplementary Figure 2C), yet no changes were detected in the atrial effects caused by the muscarinic receptor agonist, oxotremorine (0.003–3 μM) (Supplementary Figures 2A,B).

### Blockage of G protein-coupled inwardly rectifying K^+^ (GIRK/K_IR_) channels, but not of K_v_ and K_ATP_/K_IR_6 channels, counteracts atrial depression caused by A_1_ and M_2_ receptor agonists

**Figure 3:**
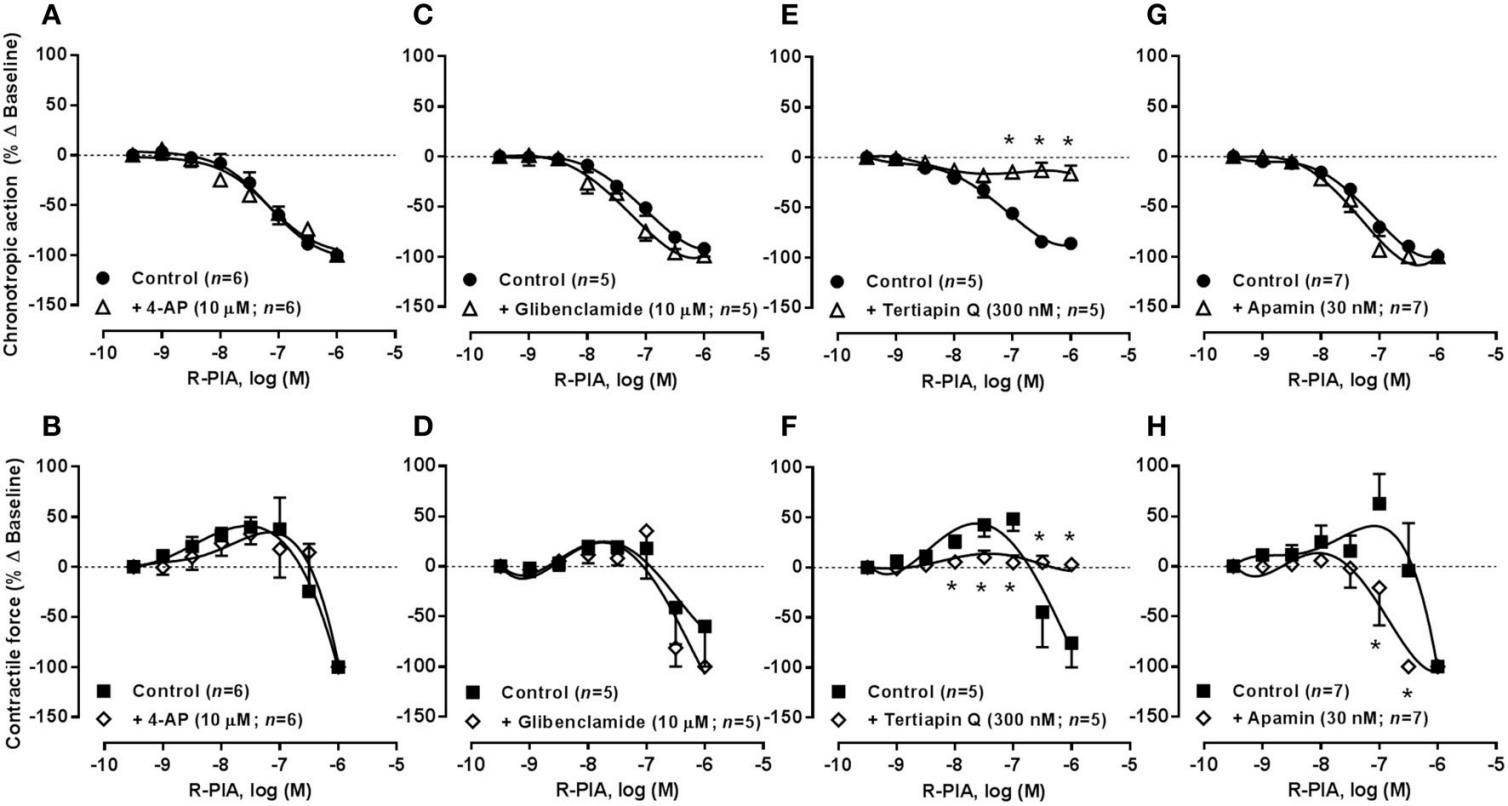
**Concentration-response curves of R-PIA (0.001–1 μM) on the spontaneously beating rat atria in the absence (Control) and in the presence of potassium channels blockers: 4-AP (10 μM, A,B), glibenclamide (10 μM, C,D), tertiapin Q (300 nM, E,F) and apamin (30 nM, G,H)**. Drug applications followed the protocol depicted in Figure 2A. The ordinates are percentage of variation of spontaneous contraction rate (chronotropic effect, **A,C,E,G**) and mechanical tension (inotropic effect, **B,D,F,H**) as compared to baseline values obtained before application of R-PIA. The data are expressed as mean ± SEM from an *n* number of individual experiments. ^*^P < 0.05 compared with the effect of R-PIA in the absence of potassium channel blockers.

**Figure 4:**
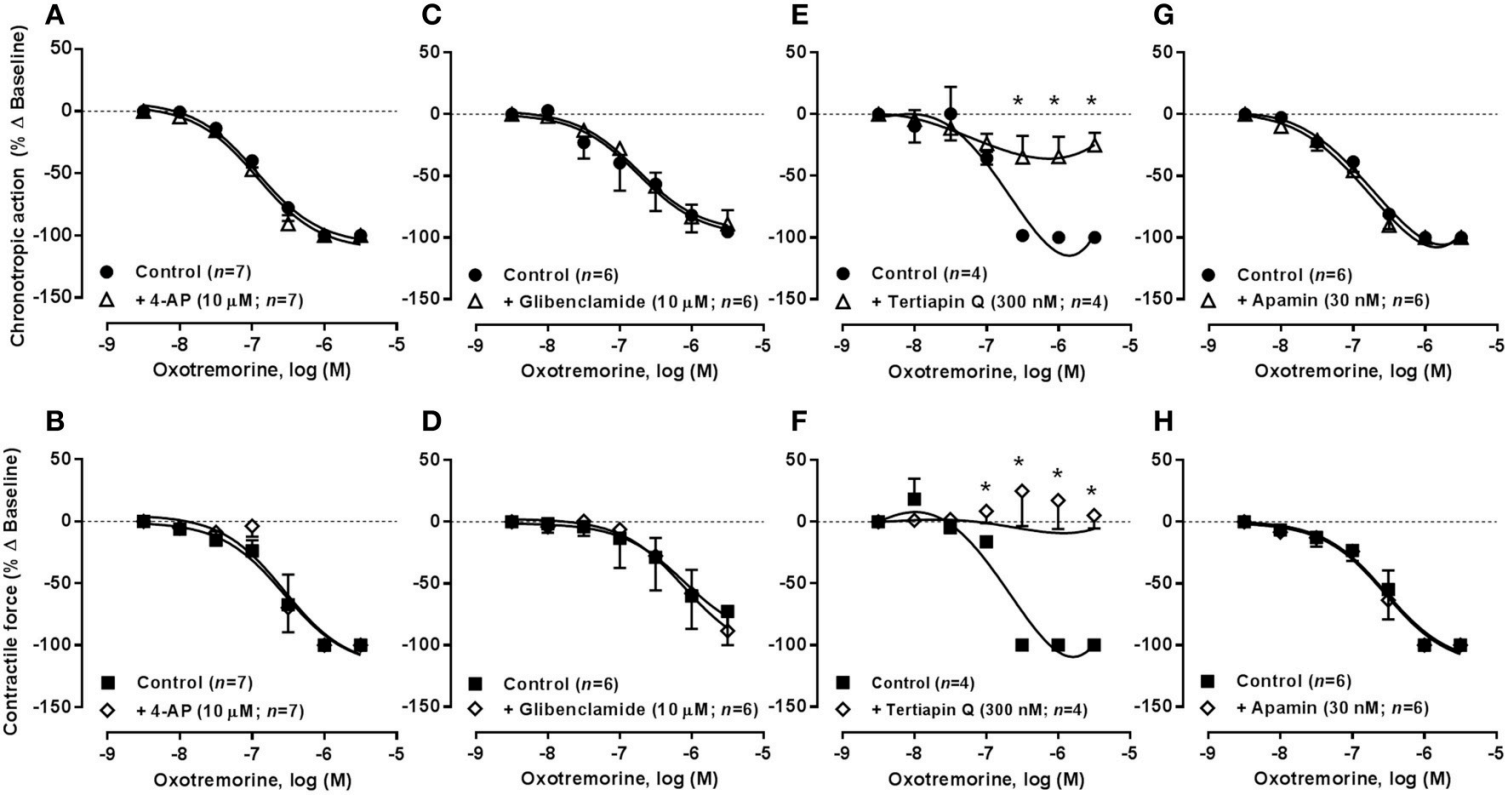
**Concentration-response curves of oxotremorine (0.003–3 μM) on the spontaneously beating rat atria in the absence (Control) and in the presence of potassium channels blockers: 4-AP (10 μM, A,B), glibenclamide (10 μM, C,D), tertiapin Q (300 nM, E,F), and apamin (30 nM, G,H)**. Drug applications followed the protocol depicted in Figure 2A. The ordinates are percentage of variation of spontaneous contraction rate (chronotropic effect, **A,C,E,G**) and mechanical tension (inotropic effect, **B,D,F,H**) as compared to baseline values obtained before application of oxotremorine. The data are expressed as mean ± SEM from an *n* number of individual experiments. ^*^P < 0.05 compared with the effect of oxotremorine in the absence of potassium channel blockers.

Figures 3 and 4 show the concentration-response curves of R-PIA (0.001–1 μM) and oxotremorine (0.003–3 μM), respectively, in spontaneously beating rat atria obtained in the absence (control) and in the presence of subtype-specific K^+^ channel blockers.

As observed in the guinea-pig atria (De Biasi et al., 1989), blockade of voltage-gated K_v_ and ATP-sensitive K_ATP_/K_IR_6 channels by 4-aminopyridine (4-AP, 10 μM) and glibenclamide (10 μM), respectively, had no significant (*P* > 0.05) effects on the negative chronotropic and inotropic actions of R-PIA and oxotremorine. Similar results were obtained when the concentration of 4-AP was increased from 10 to 100 μM (*n* = 6). On their own, 4-AP (0.01–3 mM, *n* = 5) and glibenclamide (1–100 μM, *n* = 4) increased atrial inotropism (to a maximum of 40% above control) in a concentration-dependent manner, without affecting the rate of spontaneous atrial contractions; when used at a 10 μM concentration, these inhibitors raised the force of atrial contractions by no more than 25% (see Table 2). The positive inotropic effects of 4-AP and glibenclamide were prevented by blocking β-adrenoceptors with propranolol (10 μM), agreeing with the hypothesis that these drugs may depolarize cardiac sympathetic nerve terminals favoring endogenous noradrenaline release, which may be responsible for the increase in the force of atrial contractions (data not shown).

In contrast to voltage-gated K^+^ channels, inwardly rectifier potassium channels (K_IR_) are more permeable to K^+^ during hyperpolarization than during depolarization. Activation of G protein-coupled inwardly rectifying K^+^ channels (GIRK or K_IR_3.1/3.4) by acetylcholine hyperpolarizes the resting membrane potential, thereby reducing the probability of action potential generation, while contributing to shorten atrial action potentials and the effective refractory period (ERP). Tertiapin Q (300 nM), a blocker of GIRK/K_IR_ channels with high affinity for K_IR_3.1/3.4 channels (Kodama et al., 1996; Yamada, 2002), prevented the inhibitory effects of R-PIA and oxotremorine on spontaneous atrial contractions (Figures 3D, 4D, respectively). Application of Tertiapin Q did not affect spontaneous atrial contractions when this drug was applied alone in the 0.03–1 μM concentration range (*n* = 4, see Table 2), confirming that atrial rate (chronotropic effect) and contractile force (inotropic effect) is not under the control of adenosine and acetylcholine endogenous tonus in the present experimental conditions (Han et al., 2010; but see e.g., Wang et al., 2013).

### Blockage of K_Ca_2/SK channels with apamin sensitized atria to the negative inotropic action of R-PIA, but failed to affect oxotremorine-induced atrial depression

SK channels (small conductance calcium-activated potassium channels) are a subfamily of Ca^2+^-activated K^+^ channels. Their activation limits the firing frequency of action potentials and is important for regulating after hyperpolarization in many types of electrically excitable cells. This is accomplished through the hyperpolarizing leak of positively charged potassium ions along their concentration gradient into the extracellular space. Blockade of Ca^2+^-activated K_Ca_2/SK channels with apamin (30 nM) shifted to the left the concentration-response of R-PIA (0.001–1 μM) regarding the negative inotropic component of the A_1_ receptor action (pIC_50_ 7.05 ± 0.23, *n* = 7, *P* < 0.05 vs. control) (Figure 3H), without much affecting the action of the nucleoside on atrial rate (pIC_50_ 7.48 ± 0.09, *n* = 7, *P* > 0.05 vs. control) (Figure 3G). That is, pre-treatment with apamin (30 nM) sensitized atria to the negative inotropic effect of R-PIA in a way that the negative chronotropic and inotropic actions of the A_1_ receptor agonist became evident in the same range of concentrations (pIC_50_ 7.48 ± 0.09 and 7.05 ± 0.23 for chronotropism and inotropism, respectively, *n* = 7, *P* > 0.05) (see above). Coincidently, a similar result was obtained upon reducing the K^+^ gradient across the plasma membrane by raising the extracellular concentration of KCl from 2.7 to 4.7 mM (see Supplementary Figures 2C,D). These findings suggest that the bradycardic and the negative inotropic actions of adenosine may be dissociated in terms of the intracellular mechanisms being involved. Contrariwise, apamin (30 nM), as well as changes in the extracellular concentration of KCl, failed to affect the depressant effects of oxotremorine (0.003–3 μM) on spontaneously beating rat atria under the same experimental conditions (Figures 4G,H; see also Supplementary Figures 2A,B).

**Figure 5:**
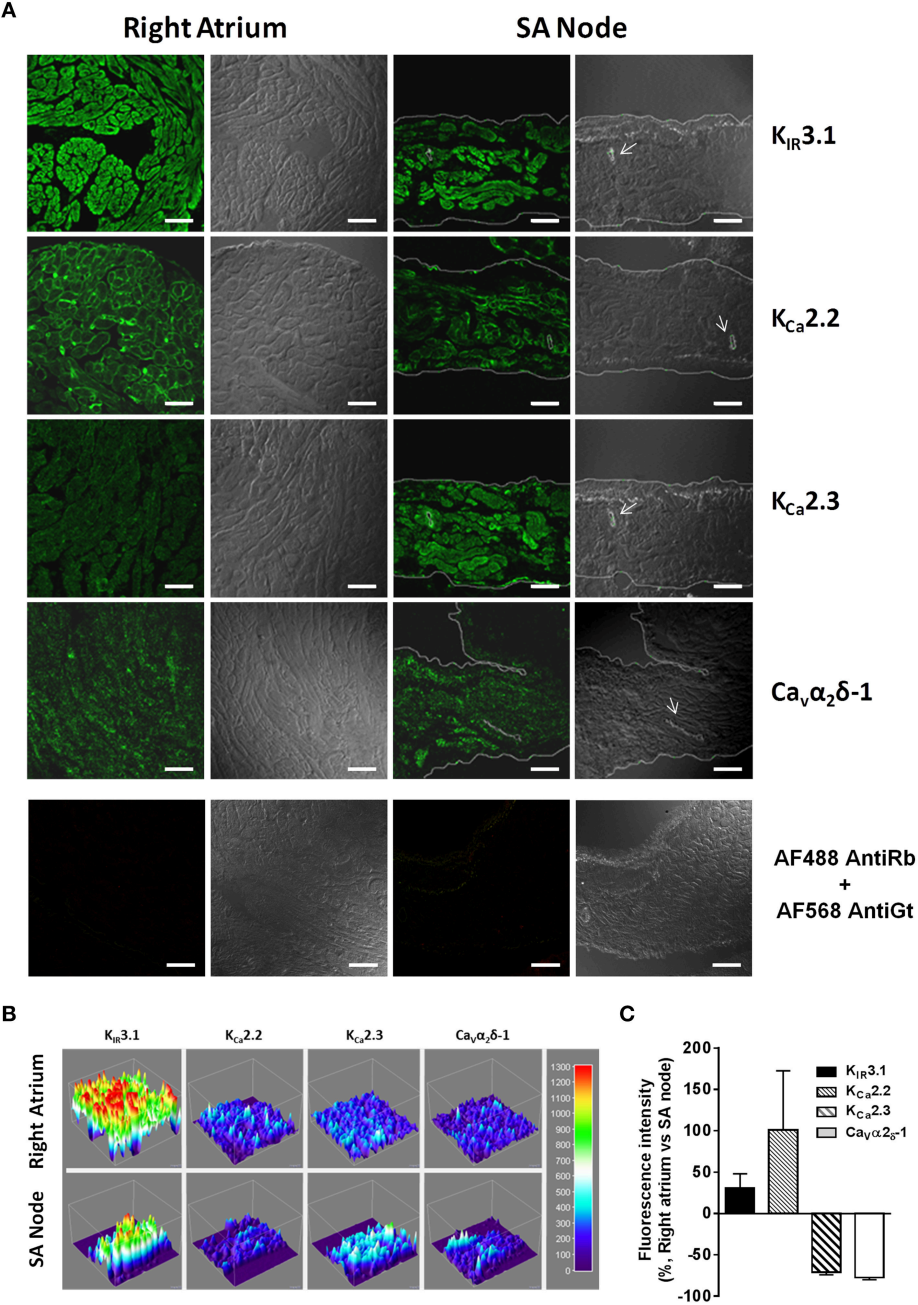
**(A)** Representative confocal micrographs of rat right atrium and SA node sections showing immunofluorescence labeling for K_IR_3.1 (GIRK1), K_Ca_2.2 (SK2), K_Ca_2.3 (SK3), and Ca_V_α_2_δ−1 channel subunits (first and third columns); the corresponding differential interference contrast (DIC) images are also shown for comparison (second and fourth columns). Last row shows cross-reactivity of secondary antibodies, Alexa Fluor 488 anti-rabbit (AF488 anti-Rb), and Alexa Fluor 568 anti-goat (AF568 anti-Gt), in which primary antibodies were omitted (see Table 3). During documentation the settings on the confocal microscope were adjusted appropriately to show immunoreactivity for sections containing both primary and secondary antibodies and these settings were maintained when documenting cross-reactivity of secondary antibodies ran in parallel to minimize biases during capture and printing of digital images. White arrows indicate SA node arteries. Similar results were obtained in five additional experiments. Horizontal bar = 30 μm. **(B)** Tridimensional surface modeling representing immunoreactivity of images depicted in panel **(A)**. Color bar represents relative fluorescence intensity map. **(C)** Graph depicting semi-quantitative analysis of K_IR_3.1, K_Ca_2.2, K_Ca_2.3, and Ca_V_α_2_δ−1 expression in right atria and SA node; the ordinates are immunofluorescence intensity ratio between K_IR_3.1, K_Ca_2.2, K_Ca_2.3, and Ca_V_α_2_δ−1 staining in paired samples from the right atrium and SA node keeping the image acquisition settings constant. Positive and negative values indicate staining predominance in contractile myocardium and SA node of the right atrium, respectively. Values are mean ± SEM; at least 3 microscopic fields were analyzed per section of the right atrium and SA node obtained from three to five rats.

Figure 5 confirms that both SA node and atrial cardiomyocytes exhibit immunoreactivity against K_IR_3.1, K_Ca_2.2, K_Ca_2.3, and Ca_v_1 channels. Differences are evident in the distribution of apamin-sensitive K_Ca_2.2 and K_Ca_2.3 channels, being the latter more abundant in the SA node compared to atrial cardiomyocytes, while the opposite was observed regarding the K_Ca_2.2 channel. High magnifications of these confocal micrographs counterstained with DAPI (nuclear dye) are shown in Supplementary Figures 3, 4. Results show that although these ion channels exhibit a mixed membranar and cytosolic pattern, ion channels specific immunostaining was absent from the nucleus. The lack of more convincing sarcolemmal staining, except for the K_Ca_2.2 channel, is a limitation of the present study. Intracellular immunostaining pattern of highly expressed plasma membrane proteins is often seen in fixed cells, as primary antibodies can enter permeabilized cells and bind to target proteins localized in the Golgi during their trafficking to the plasma membrane or in caveolae and/or endosomes when subject to recycling from the plasma membrane, as part of membrane plasticity phenomena. Fluorescent immunostaining of receptors and ion channels may also be differently distributed at the periphery of myocytes and in transverse tubule (T-tubule) invaginations of the sarcolemmal membrane, both in living and permeabilized cardiomyocytes (Balijepalli et al., 2003). Prominent T-tubule-staining pattern can be recognized as fine fluorescent spots inside cardiac myocytes. The functional significance of intracellular localization of plasma membrane receptors and ion channels certainly deserves future investigations, which are beyond the scope of this study.

### Isolated atrial cardiomyocytes exhibit a delayed outward K^+^ current that is dependent on Ca^2+^ influx through Ca_v_1 (L-Type) channels

**Figure 6:**
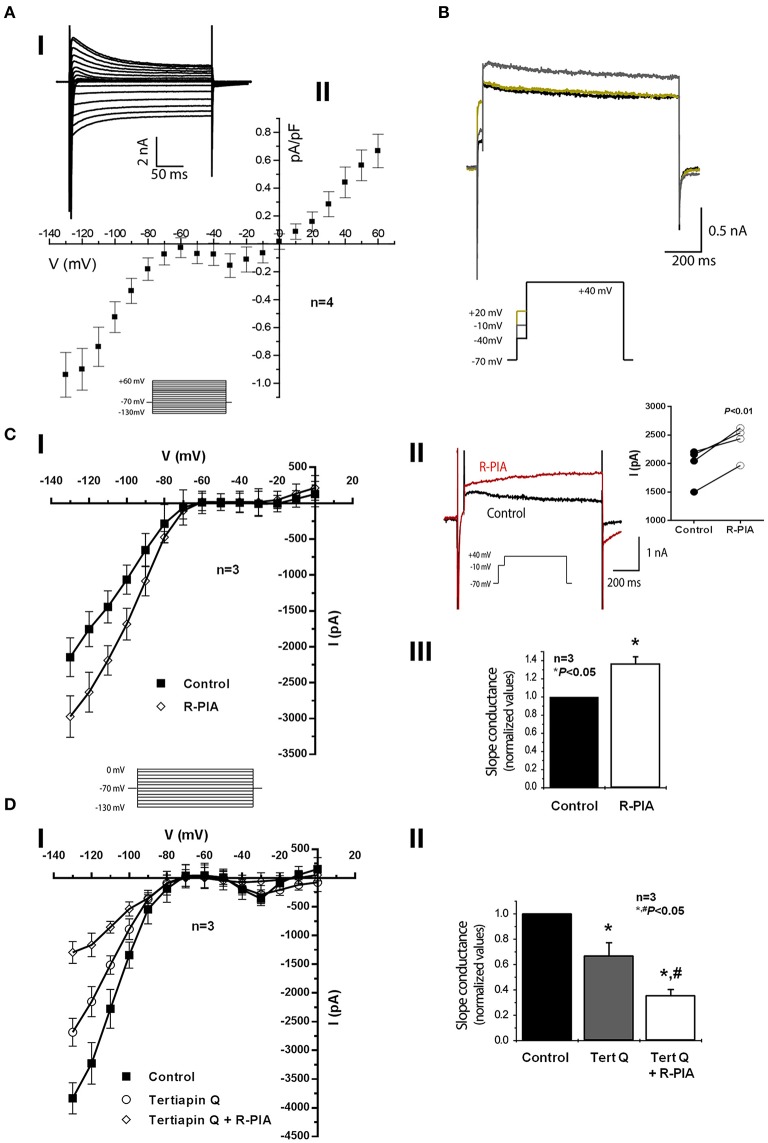
**Effects of adenosine A_1_ receptor activation on whole-cell voltage-clamp recordings in rat atrial myocytes. (Ai)** Representative currents following a set of voltage pulses (260 ms), covering a wide range of potentials, with incremental depolarization steps (10 mV) from −130 to +60 mV (holding voltage −70 mV) (see inset). **(Aii)** Corresponding current density-voltage relationship showing three different components in terms of voltage dependence: a strong inward rectifier, an inward “hump,” and a delayed outward current. Data are expressed as mean ± SEM of four animals; recordings from three to five isolated atrial cardiomyocytes were averaged per experimental animal. **(B)** Representative current traces from a triple set of double depolarizing pulses: one first step to −40, −10, and +20 mV, lasting 50 ms, followed by a second pulse to +40 mV, lasting 750-ms (see inset). One can notice a larger outward current at +40 mV when preceded by a prepulse to −10 mV, which suggests a Ca^2+^-dependent current component. Panels **(C,D)** show current-voltage relationships obtained from currents recorded following a set of voltage steps (−130 to 0 mV, 10 mV steps, holding voltage −70 mV, 260 ms duration each) in the absence (Control) and in the presence of R-PIA (300 nM, **Ci**) and tertiapin Q (300 nM, **Di**) with or without R-PIA (300 nM). Data are expressed as mean ± SEM of three animals; recordings from four to five isolated atrial cardiomyocytes were averaged per experimental animal. Panel **(Cii)** shown are typical recording traces showing that activation of the adenosine A_1_ receptor with R-PIA (300 nM) increases the Ca^2+^-dependent outward current obtained following application of the double-pulse protocol consisting of one pre-pulse of 50 ms duration to −10 mV immediately followed by a second pulse to +40 mV lasting 750-ms (see inset). This experiment was repeated using four cardiomyocytes isolated from three different animals (right-hand side panel); ^*^P < 0.01 (paired Student's *t*-test) represent significant differences from control. **(Ciii)** Refers to normalized values of slope conductance (calculated with measurements of −120 to −80 mV) in cells obtained from three different animals in the absence (Control) and in the presence of R-PIA (300 nM). Panel **(Dii)** shows similar experiments as **(Ciii)**, but in this case tertiapin Q (300 nM) with or without R-PIA (300 nM) was used instead of R-PIA alone. Error bars represent SEM of three animals. ^*^,^#^*P* < 0.05 (unpaired Student's *t*-test with Welch's correction) represent significant differences from control or from tertiapin Q alone, respectively.

The functionality of small conductance Ca^2+^-activated K_Ca_2/SK outward channels was tested by performing voltage-clamp experiments in acutely dissociated rat atrial cardiomyocytes using the whole-cell patch-clamp configuration. To evaluate the current-voltage dependence, we covered a wide range of potentials by setting 10 mV steps (of 260 ms) from −130 to +60 mV, while keeping the holding potential at −70 mV (Figure 6A). The corresponding voltage-intensity curves revealed three main components (Figures 6Ai,ii). An obvious inward rectifying component was resolved in the range of −130 to −70 mV. From −50 to 0 mV, we detected an inward “hump,” whereas a typical delayed outward K^+^ current was observed above +10 mV. Unfortunately, this protocol could only be applied a limited number of times, as patch seals tend to break down due to contraction of cardiomyocytes particularly at the most depolarized steps, thus precluding further pharmacological manipulations. The use of cardioplegic drugs, such as 2,3-butanedione monoxime, after the isolation procedure was out of the question as it affects several ion channels. Lowering Ca^2+^ beyond a certain threshold and/or increasing the cell Ca^2+^ buffering capacity was also disadvantageous because it could preclude investigation of Ca^2+^-activated K^+^ currents. Hence, to investigate outward K^+^ currents over long periods of time as required in this study (see below), a less abrasive single voltage step (to +40 mV) was performed (Figure 6B). This protocol consisted in a brief depolarization (50 ms) to −10 mV aiming at Ca^2+^ influx through voltage-activated channels, which was immediately followed by a longer depolarizing step (750 ms) to +40 mV. This was used because the resolution of the outward current was greater when preceded by a brief depolarizing step to −10 mV as compared to the situation where the voltage of preceding pulses was raised to −40 mV or to +20 mV; the same trend was observed in cells from three different animals. Moreover, the outward K^+^ current recorded at +40 mV was greater if the duration of the prepulse (to −10 mV) was increased (data not shown; see e.g., Marrion and Tavalin, 1998).

The inward current “hump” detected in the current-voltage relationship peaking at −30 and −10 mV (Figure 6A) suggests the activation of voltage-gated calcium channels allowing Ca^2+^ influx mainly through high-voltage Ca_v_1 (L-type) channels (Grant, 2009). Even though we did not perform experiments to directly evaluate calcium dynamics, it is likely that activation of voltage-activated calcium channels may account for the inward current “hump” observed here. Thus, Ca^2+^ recruitment through voltage-gated channels, most probably via high-voltage Ca_v_1 (L-type) channels, ensures coupling to small-conductance Ca^2+^-activated K^+^ outward currents to occur as described in central neurons (Marrion and Tavalin, 1998) and, most importantly, in cardiac myocytes (Lu et al., 2007). In an attempt to elucidate the differential shape of action potentials between atrial and ventricular myocytes, Baro and Escande (1989) found long lasting Ca^2+^-activated K^+^ outward currents in atrial myocytes, which were sensitive to apamin. Consistent with this report, we show here that the outward K^+^ current was clearly voltage-dependent with the maximal amplitude obtained when atrial cardiomyocytes were depolarized with a prepulse reaching −10 mV, i.e., close to the maximum activation of high-voltage Ca_v_1 (L-type) currents.

### The adenosine A_1_ receptor plays a dual role in atrial cardiomyocytes by activating GIRK/K_IR_3.1/K_IR_3.4 and by inhibiting K_Ca_2/SK mediated outward currents depending on cell depolarization

Incubation of isolated atrial cardiomyocytes with the adenosine A_1_ receptor agonist, R-PIA (300 nM), increased the magnitude of the outward K^+^ current (Figure 6Ci) by increasing the slope conductance 1.36 fold above the control value (Figure 6Ciii). Figure 6Cii shows that R-PIA (300 nM) further increased the magnitude of the outward current when this was preceded by a brief (50 ms) depolarizing step to −10 mV allowing Ca^2+^ influx through high-voltage Ca_v_1 (L-type) channels (Grant, 2009); this trend was observed in four out of seven cells from three different animals (right-hand side panel), but the magnitude of the outward current in the presence of R-PIA varied considerably reflecting heterogeneity of the cells that compose atrial muscle. Interestingly, R-PIA (300 nM) changed significantly the kinetics of the outward current recorded at +40 mV (Figure 6Cii).

**Figure 7:**
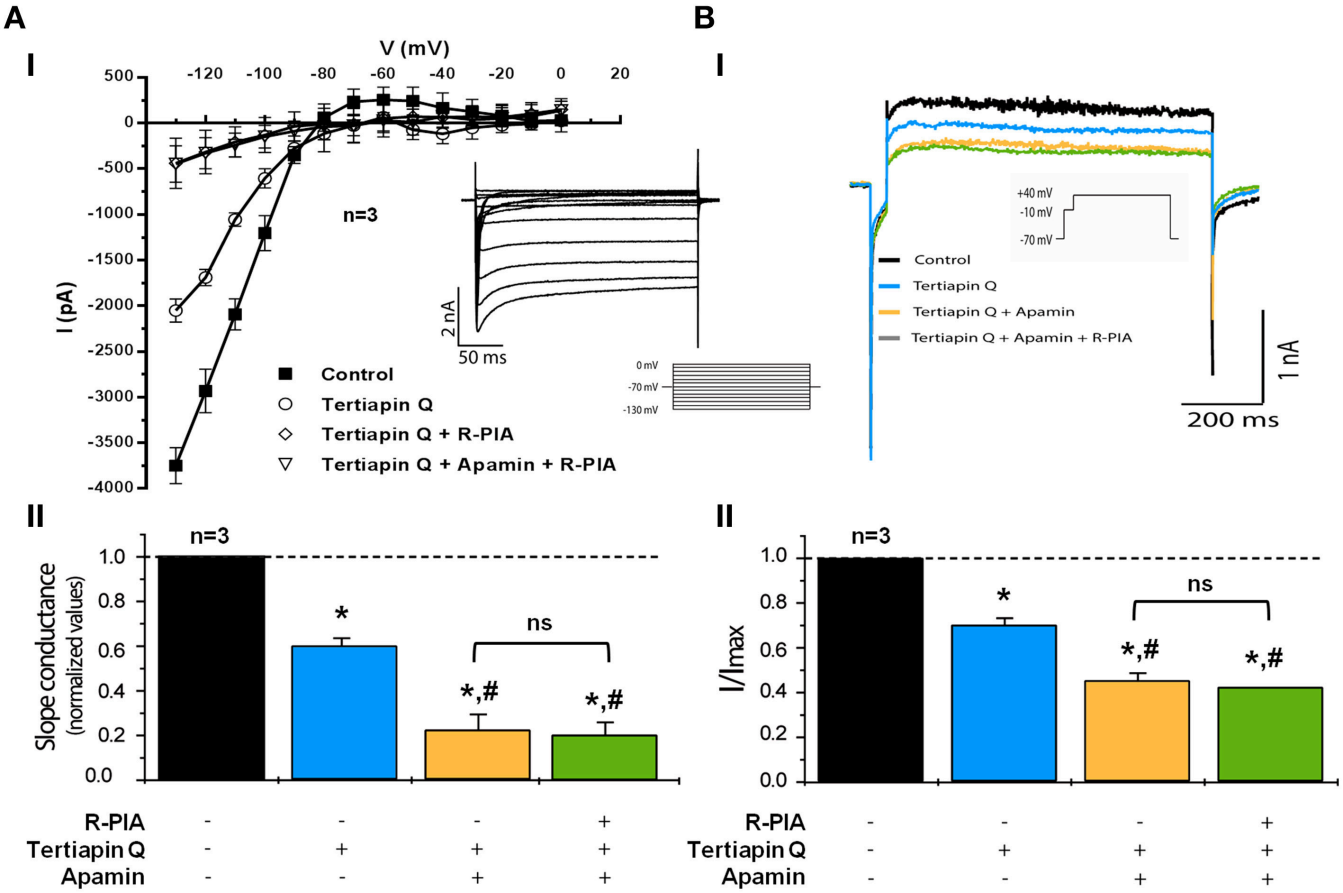
**Activation of adenosine A_1_ receptors inhibits Ca^2+^-activated outward K_Ca_2/SK current in isolated rat atrial myocytes when inwardly rectifying GIRK/K_IR_3 channels are blocked with tertiapin Q. (Ai)** Current-voltage relationship showing strong inward rectification obtained from currents recorded following a set of voltage steps (−130 to 0 mV, 10 mV steps, holding voltage −70 mV, 260 ms duration each) in the absence (Control) and in the presence of tertiapin Q (300 nM), apamin (30 nM) and R-PIA (300 nM), applied cumulatively (see the inset for representative currents). Data are expressed as mean ± SEM of three different animals; recordings from three to five isolated atrial cardiomyocytes were averaged per experimental animal. **(Aii)** Bar-graphs representing pooled data from three different animals in which the average slope conductance of the inward current was normalized to the maximum obtained in the control situation, without test drugs. In panel **(B)** shown are representative whole-cell voltage-clamp recording traces from double depolarization protocol held at holding potential of −70 mV using the same cell as in **(Ai)**. Currents were elicited by a single 10 ms depolarizing pulse from −70 to −10 mV followed by a 750-ms pulse to +40 mV (see inset in panel **Bi**). Panel **(Bi)** shows the effects on Ca^2+^-activated outward K_Ca_2/SK currents of cumulative applications of tertiapin Q (300 nM), apamin (30 nM), and R-PIA (300 nM). Sodium currents were truncated to facilitate visualization of effects. **(Bii)** Bar-graphs representing pooled data from three similar experiments in which peak current was normalized to the maximum current obtained in the absence of added drugs (Control). All experiments were performed in the presence of E4031 (10 μM) to prevent rapid delayed rectifier potassium currents operated by hERG channels from being activated. Error bars represent SEM of three animals. ^*^, ^#^*P* < 0.05 (unpaired Student's *t*-test with Welch's correction) represent significant differences from the control or from tertiapin Q alone, respectively; ns, not significant.

Stimulation of A_1_ receptors in atrial myocytes is known to activate inwardly-rectifying GIRK/K_IR_3.1/K_IR_3.4 channels (Belardinelli and Isenberg, 1983; Kurachi et al., 1986; Yatani et al., 1987; Mubagwa and Flameng, 2001), which may lead to the appearance of a slowly activating component in the outward current (Belardinelli and Isenberg, 1983; Kurachi et al., 1986; Banach et al., 1993; Wellner-Kienitz et al., 2000; Lomax et al., 2003). To test this hypothesis, we investigated the effect of the adenosine A_1_ receptor agonist, R-PIA (300 nM), under blockage of GIRK/K_IR_3.1/K_IR_3.4 channels. Pretreatment with tertiapin Q (300 nM) decreased the whole-cell conductance (Figures 6D, 7A). More relevant to the present context, subsequent activation of A_1_ receptors with R-PIA (300 nM), still in the presence of tertiapin Q (300 nM), caused a further decrease, instead of an increase, of the whole-cell outward current (Figures 6Di,ii). This suggests that upon activation of the adenosine A_1_ receptor, two potassium currents are altered. While the inwardly-rectifying GIRK/K_IR_3.1/K_IR_3.4 current is enhanced, a second current subsisting after blockage of GIRK/K_IR_3.1/K_IR_3.4 channels with tertiapin Q is inhibited by the A_1_ receptor agonist.

We, then, set up to investigate the nature of the R-PIA-sensitive outward current observed under conditions of GIRK/K_IR_3.1/K_IR_3.4 channel blockade (Figure 7). In the presence of tertiapin Q (300 nM), application of apamin (30 nM) to the superfusion fluid decreased further the Ca^2+^-dependent delayed outward K^+^ current (Figure 7B). At 30 nM concentration the current mediated by small conductance Ca^2+^-activated K_Ca_2/SK channels are supposed to be substantially blocked by apamin (Xu et al., 2003; Tuteja et al., 2005). Under these pharmacological restraining conditions, subsequent application of R-PIA (300 nM) did not cause further modifications (*P* > 0.10) in the outward current (Figure 7B). Overall, these results show that (1) there is a component of the outward current that is mediated by small conductance Ca^2+^-activated K_Ca_2/SK channels, and (2) such K_Ca_2/SK component is not modified further by A_1_ receptors activation, since R-PIA failed to alter the whole-cell current in the presence of apamin (30 nM).

Figure 7A shows data obtained when evaluating whole-cell conductance using a similar pharmacological approach. The reduction in the magnitude of the inwardly-rectifying current caused by tertiapin Q (300 nM) was amplified by blocking K_Ca_2/SK channels with apamin (30 nM). Notably, blockade of GIRK/K_IR_3.1/K_IR_3.4 and K_Ca_2/SK channels with tertiapin Q (300 nM) and apamin (30 nM), respectively, prevented further changes in whole-cells conductance by application of the A_1_ receptor agonist, R-PIA (300 nM) (Figures 7A,B). Thus, electrophysiological data strongly indicate that besides activation of inwardly rectifying GIRK/K_IR_3.1/K_IR_3.4 currents (Belardinelli and Isenberg, 1983; Kurachi et al., 1986; Yatani et al., 1987; Mubagwa and Flameng, 2001), stimulation of adenosine A_1_ receptors leads to inhibition of apamin-sensitive Ca^2+^-activated K_Ca_2/SK channels in atrial cardiomyocytes.

### Loss of atrial chronoselectivity of adenosine A_1_ receptors by blocking high voltage-activated Ca_v_1 (L-Type) currents with nifedipine or verapamil

**Figure 8:**
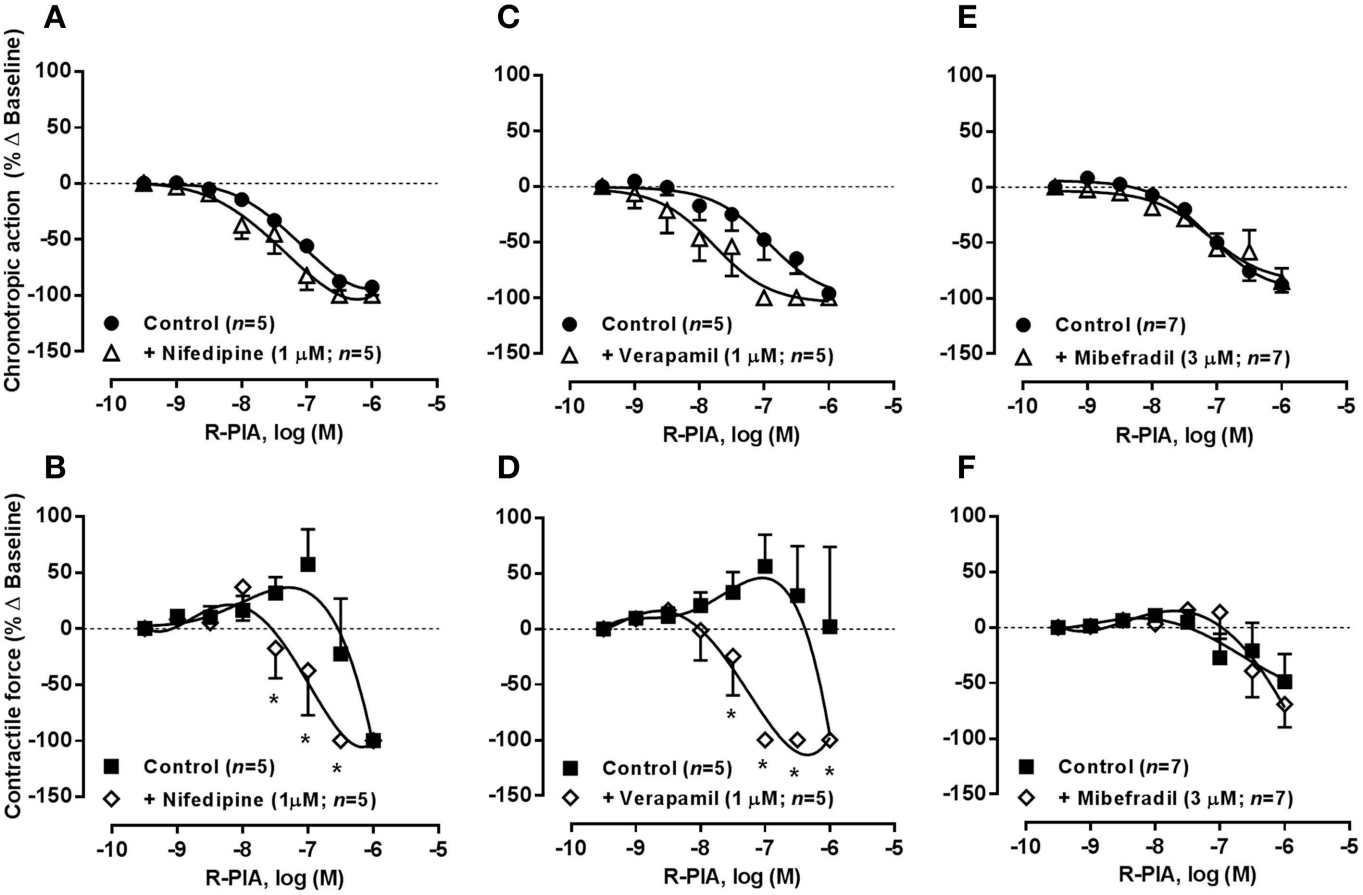
**Concentration-response curves of R-PIA (0.001–1 μM) on the spontaneously beating rat atria in the absence (Control) and in the presence of voltage-sensitive calcium channels inhibitors: nifedipine (1 μM, A,B), verapamil (1 μM, C,D), and mibefradil (3 μM, E,F)**. Drug applications followed the protocol depicted in Figure 2A. The ordinates are percentage of variation of spontaneous contraction rate (chronotropic effect, **A,C,E**) and mechanical tension (inotropic effect, **B,D,F**) as compared to baseline values obtained before application of R-PIA. The data are expressed as mean ± SEM from an *n* number of individual experiments. ^*^P < 0.05 compared with the effect of R-PIA in the absence of voltage-sensitive calcium channels inhibitors.

**Figure 9:**
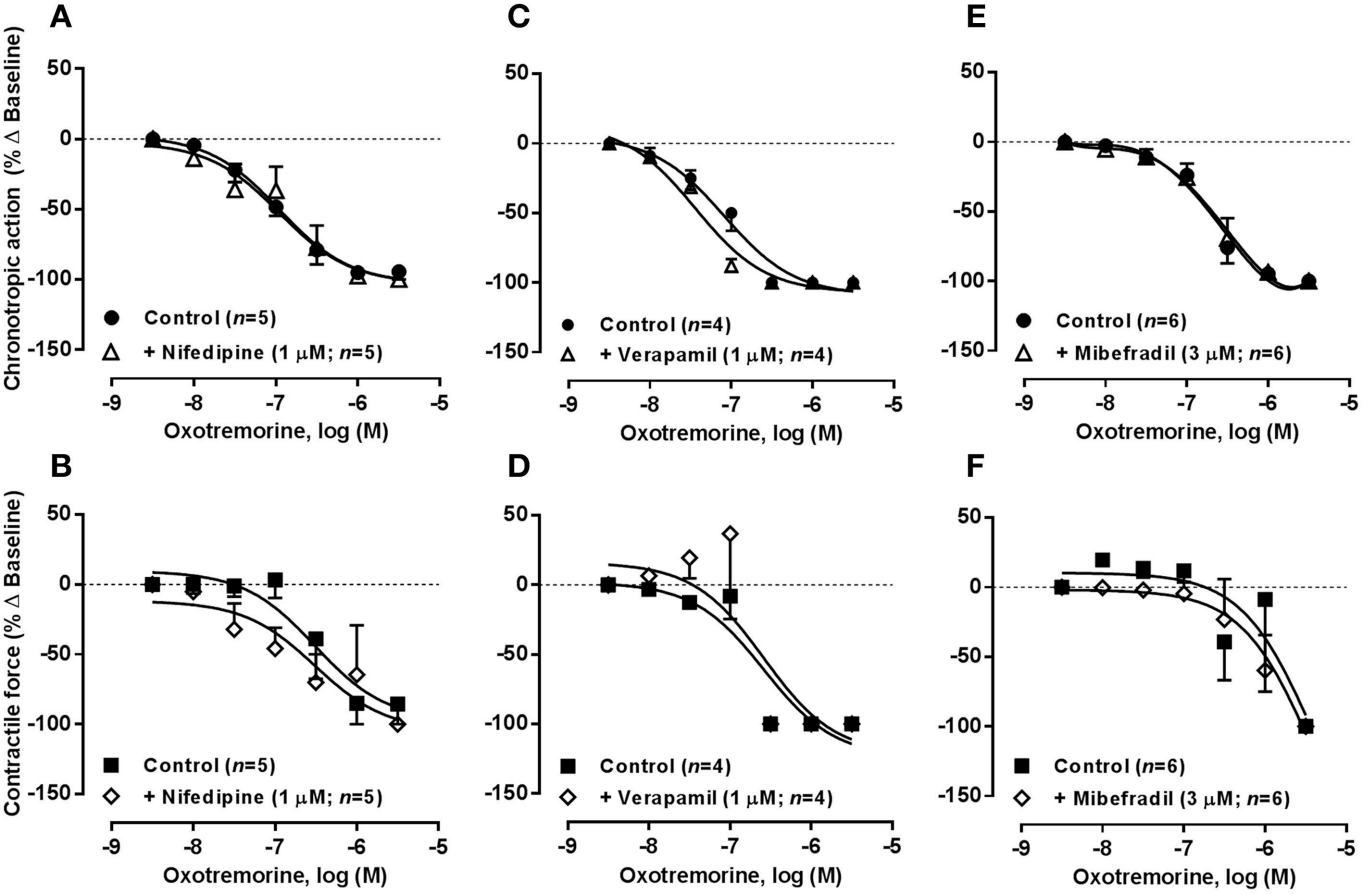
**Concentration-response curves of oxotremorine (0.003–3 μM) on the spontaneously beating rat atria in the absence (Control) and in the presence of voltage-sensitive calcium channels inhibitors: nifedipine (1 μM, A,B), verapamil (1 μM, C,D), and mibefradil (3 μM, E,F)**. Drug applications followed the protocol depicted in Figure 2A. The ordinates are percentage of variation of spontaneous contraction rate (chronotropic effect, **A,C,E**) and mechanical tension (inotropic effect, **B,D,F**) as compared to baseline values obtained before application of oxotremorine. The data are expressed as mean ± SEM from an *n* number of individual experiments.

The K_Ca_2/SK channel gating mechanism is controlled by intracellular Ca^2+^ levels entering via voltage-activated calcium channels (Ca_v_) (Marrion and Tavalin, 1998). Figure 8 shows that blockade of Ca_v_1 (L-type) channels with nifedipine or verapamil, applied in a concentration (1 μM) that reduced atrial chronotropy roughly by 25% (see Table 2), sensitized atria to the negative inotropic action of R-PIA (0.001–1 μM) without much affecting the action of the A_1_ receptor agonist on atrial rate. The negative chronotropic and inotropic actions of R-PIA were both evident at the same concentration range in the presence of Ca_v_1 (L-type) channel blockers, nifedipine (pIC_50_ 7.51 ± 0.10 and 7.14 ± 0.14 for chronotropism and inotropism, respectively, *n* = 5, *P* > 0.05) or verapamil (pIC_50_ 7.80 ± 0.20 and 7.34 ± 0.24 for chronotropism and inotropism, respectively, *n* = 5, *P* > 0.05), thus resulting in a loss of the atrial chronoselectivity of adenosine A_1_ receptors (see above). Under similar experimental conditions, pre-treatment with acetylcholine did not significantly (*P* > 0.05) modify the depressing effects of R-PIA (0.001–1 μM) on spontaneously beating rat atria, when the cholinergic agonist was applied in a concentration (30 μM) that mimicked the negative chronotropic effect (25% reduction from control, *n* = 3–4) of nifedipine or verapamil. Conversely, blockade Ca_v_1 (L-type) channels with 1 μM nifedipine or verapamil failed to modify oxotremorine (0.003–3 μM)-induced depression in the rat spontaneously beating atria (Figure 9). These findings contrast with those obtained with the adenosine A_1_ receptor agonist, suggesting that the negative chronotropic and inotropic actions of oxotremorine are independent on Ca^2+^ influx through Ca_v_1 (L-type) channels and on apamin-sensitive K_Ca_2/SK channels, relying most probably on the control of K^+^ currents via G protein-coupled inwardly rectifying K^+^ channels (GIRK or K_IR_3.1/3.4) sensitive to tertiapin Q.

Blockade of low voltage-activated Ca_v_3 (T-type) channels with mibefradil (0.1–10 μM) concentration-dependently reduced atrial chronotropy to a maximum of 30% (*n* = 7), without affecting the force of spontaneous atrial contractions (see Table 2). Mibefradil (3 μM) failed to modify the effects of both R-PIA (0.001–1 μM, Figures 8E,F) and oxotremorine (0.003–3 μM, Figures 9E,F) in the spontaneously beating rat atria (see also Belardinelli et al., 1995).

### Interplay between K_Ca_2/SK, GIRK/K_IR_, and Ca_v_1 (L Type) channels on spontaneously beating rat atria

**Figure 10:**
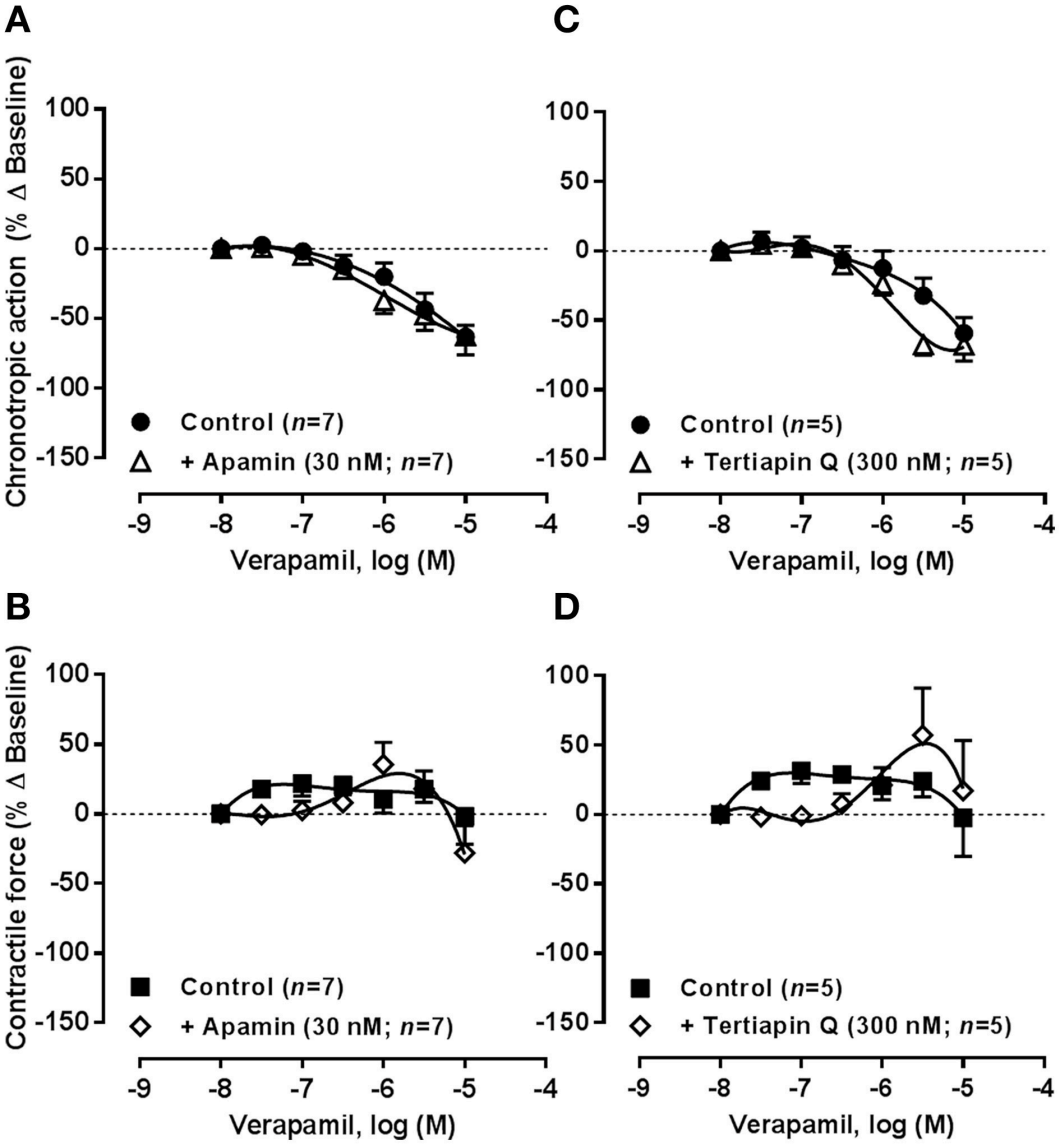
**Concentration-response curves of the Ca_v_1 (L-type) channel inhibitor, verapamil (0.03–10 μM), on the spontaneously beating rat atria in the absence (Control) and in the presence of apamin (30 nM, A,B) and tertiapin Q (300 nM, C,D), which selectively block K_Ca_2/SK and GIRK/K_IR_ channels, respectively**. Drug applications followed the protocol depicted in Figure 2A. The ordinates are percentage of variation of spontaneous contraction rate (chronotropic effect, **A,C**) and mechanical tension (inotropic effect, **B,D**) as compared to baseline values obtained before application of verapamil. The data are expressed as mean ± SEM from an *n* number of individual experiments.

Figure 10 shows the responses of spontaneously beating rat atria to the cumulative application of verapamil (0.03–10 μM) in the absence and presence of apamin (30 nM) or tertiapin Q (300 nM). Verapamil (0.03–10 μM), as well as nifedipine (0.03–30 μM, data not shown), decreased the rate of contractions of the rat spontaneously beating atria in a concentration-dependent manner. Similarly to adenosine A_1_ receptor agonist, the negative chronotropic effect of verapamil (0.03–10 μM, Figures 10A,C) was evidenced in concentrations unable to decrease the magnitude of atrial tension (Figures 10B,D). It is worth noting that the negative inotropic potency of R-PIA (Figure 2C) and verapamil (data not shown) was not significantly (*P* > 0.05) modified in atria paced electrically at a constant frequency of 240 beats.min^−1^; for instance, the pIC_50_ values obtained for the negative inotropic action of R-PIA were not different (*P* > 0.05) in spontaneously beating (6.14 ± 0.07, *n* = 38) and electrically-paced (6.20 ± 0.05, *n* = 7) rat atria. A similar situation was detected regarding the negative inotropic effect of oxotremorine in spontaneously beating (pIC_50_ value of 6.81 ± 0.10, *n* = 31) and electrically-paced (pIC_50_ value of 7.22 ± 0.15, *n* = 7) atria (Figure 2B). These findings were obtained notwithstanding the maximal reduction of myographic recordings amplitude induced by pacing in the presence of R-PIA and oxotremorine did not go beyond 30% of that observed in spontaneously beating atria (see Figure 2B). Thus, data indicate that sustained inotropy is not a direct consequence of the intracellular residual Ca^2+^ accumulation due to slowing down the rhythm of atrial contractions (negative dromotropic/chronotropic effects) in response to drug applications. However, in contrast to the adenosine A_1_ receptor agonist, blockade of K_Ca_2/SK and GIRK/K_IR_ channels respectively with apamin (30 nM) and tertiapin Q (300 nM) failed to modify atrial effects produced by verapamil (0.03–10 μM).

**Figure 11:**
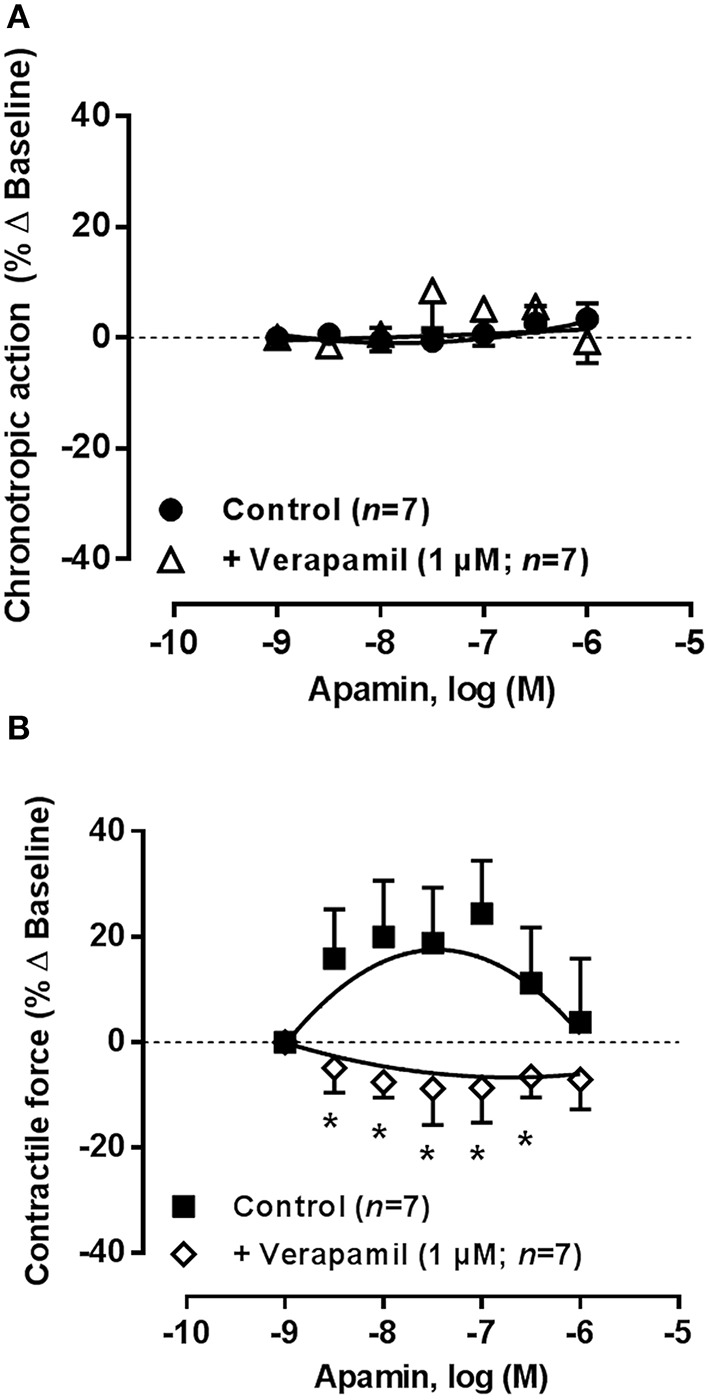
**Concentration-response curves of the K_Ca_2/SK channel blocker, apamin (0.003–1 μM), on the spontaneously beating rat atria in the absence (Control), and in the presence of verapamil (1 μM)**. Drug applications followed the protocol depicted in Figure 2A. The ordinates are percentage of variation of spontaneous contraction rate (chronotropic effect, **A**) and mechanical tension (inotropic effect, **B**) as compared to baseline values obtained before application of apamin. The data are expressed as mean ± SEM from an *n* number of individual experiments. ^*^P < 0.05 compared with the effect of apamin in the absence of verapamil.

In another set of experiments, we tested the effects of increasing concentrations of apamin (0.003–1 μM) in the absence and in the presence of verapamil (1 μM) (Figure 11). Blockade of Ca^2+^-activated K_Ca_2/SK channels with apamin (0.003–1 μM) did not significantly (*P* > 0.05) change the spontaneous atrial rate (Figure 11A), but we observed a moderate positive inotropic effect (maximal increase ~20% at 0.1 μM, Figure 11B) in line with that occurring with other K^+^ channel blockers, 4-AP and glibenclamide (see above). Interestingly, the mild positive inotropic effect of apamin (0.003–1 μM) reproduced the transient increase in atrial contractile force caused by the adenosine A_1_ receptor agonist, R-PIA, in a wide range of concentrations (1–100 nM, see Figure 2), but the K_Ca_2/SK channel inhibitor had no comparable effect on chronotropy. Verapamil (1 μM) reversed the positive inotropic effect of apamin leading to a mild negative inotropic effect (Figure 11B). Data suggest that strengthening spontaneous atrial contractions by blocking K_Ca_2/SK channels requires Ca^2+^ influx through verapamil-sensitive Ca_v_1 (L type) channels.

## Discussion

The present study demonstrates that adenosine and its stable analog, R-PIA, acting via inhibitory A_1_ receptors are chronoselective atrial depressants affecting inotropy in a much lesser degree compared to the muscarinic M_2_ receptor agonist, oxotremorine, in spontaneously beating rat atria. Likewise, intravenous R-PIA caused a marked and sustained sinus bradycardia without affecting myocardial contractility leading to a reduction in the mean arterial blood pressure in anesthetized pigs (Wainwright and Parratt, 1993). A different result was obtained by Lee et al. (1993), since the A_1_ receptor agonist, N^6^-cyclopentyladenosine (CPA), was about equipotent on electrically-driven left atria (negative inotropic action) and on spontaneously beating right atria (negative chronotropic action). However, these authors used a pacing frequency of 1 Hz (60 beats.min^−1^) that is far too low compared to the spontaneous atrial rate (200–220 beats.min^−1^) as verified in our study using both right and left atria with the SA node intact (see Table 2). We show here that activation of the A_1_ receptor with R-PIA decreases the contractile force of atria paced at 4 Hz frequency (just slightly above the spontaneous atrial rate) by a similar extent to that observed in spontaneously beating atria. In anesthetized pigs, right atrial pacing with a frequency just above the spontaneous sinus rate in the pig (110 beats.min^−1^) abrogated R-PIA-induced decrease in blood pressure, showing that the fall in blood pressure seen in unpaced animals was a result of decreased cardiac rate and was not due to a reduction in myocardial contractility and/or to peripheral vasodilatation. These findings indicate that negative chronotropic-dependent inotropy, which has been pointed out as a major drawback of experiments performed in spontaneously beating isolated atria (Stemmer and Akera, 1986), does not account significantly to adenosine chronoselectivity. Supersensitive negative chronotropic and dromotropic effects of adenosine have been described in the presence of isoproterenol in the transplanted human heart in which adenosine failed to attenuate the isoproterenol-induced increase in contractility whereas it produced an exaggerated negative chronotropic and dromotropic effect (Koglin et al., 1996). Isolated spontaneously beating atria as used in the present study are devoid of counterregulatory inotropic actions of the sympathetic nervous system. A remaining stimulation of β_1_-adrenoceptors can also not afford a valid explanation for adenosine chronoselectivity in our study since atria were unaffected by the application of propranolol (Dobson, 1983; Romano et al., 1991).

Immunofluorescence confocal microscopy showed no significant differences in the distribution of the A_1_ receptor protein between SA node and atrial cardiomyocytes suggesting that it can also not afford a rationale for adenosine chronoselectivity (see Figure 1D). This was observed despite the demonstration that the A_1_ receptor mRNA expression was higher in the right atrium than in the SA node, with no differences found on M_2_ receptor mRNA levels between distinct atrial regions (Chandler et al., 2009). These findings suggest that the negative chronotropic and inotropic atrial responses to adenosine, via A_1_ receptors, are differentiated at the postreceptor transduction level (see e.g., Oguchi et al., 1995). While both A_1_ and M_2_ receptors promote K^+^ efflux through βγ subunits of G protein-coupled inwardly rectifying (tertiapin Q-sensitive) GIRK/K_IR_3 channels modulating SA node automatism (Belardinelli and Isenberg, 1983; Kurachi et al., 1986; Yatani et al., 1987; Mubagwa and Flameng, 2001), we demonstrated here for the first time that activation of adenosine A_1_ receptors concurrently inhibit small conductance outward K_Ca_2/SK currents probably via G protein α subunit.

Cardiac K^+^ channels have been recognized as potential targets for the actions of neurotransmitters, hormones and class III antiarrhythmic drugs that prolong action potential duration and refractoriness; potassium channel inhibitors can effectively prevent/inhibit cardiac arrhythmias (Li and Dong, 2010). Among all K^+^ channel inhibitors used in this study, namely 4-aminopyridine, glibenclamide, and apamin, only the selective GIRK/K_IR_3.1/3.4 channel blocker, tertiapin Q, produced no significant effects on spontaneously beating atria when used alone. It is, therefore, reasonable to conclude that the primary effect of tertiapin Q on spontaneously beating atria is limited to blockade of muscarinic- and adenosine-activated GIRK/K_IR_3.1/3.4 currents and that, in the absence of endogenous ligands, both atrial rate and contractile force are not controlled by constitutive A_1_ and M_2_ receptors activity. Scarcity of intrinsic A_1_ receptor tone in the absence of endogenous adenosine was also inferred from the lack of effect of DPCPX (2.5–100 nM) alone on spontaneous atrial performance, as this compound has inverse agonist properties in systems with constitutive A_1_ receptor activity (Searl and Silinsky, 2012; He et al., 2013). Besides tertiapin Q, the repercussion of K^+^ channels blockade on atrial activity may reflect a positive inotropic effect operated either directly due to cardiomyocyte depolarization, or indirectly by promoting noradrenaline release from depolarized sympathetic nerves associated to blockage of outward K^+^ currents at resting membrane potential (Lawson, 1996; Xu et al., 2003; Tuteja et al., 2005; Grant, 2009). The participation of noradrenaline release from sympathetic nerves was assumed because the inotropic effect of K^+^ channel inhibitors was prevented by the β-receptor blocker, propranolol.

Moreover, the resting heart rate in unrestrained conscious wild-type and GIRK4 knockout mice was virtually identical, indicating that other signaling pathways involved in heart rate regulation might balance the missing I_KACh_ current (Wickman et al., 1998). These authors showed that the diminished bradycardic response of the GIRK4 knockout mice to A_1_ receptor activation was only fifty percent of the heart rate decrease in response to adenosine and acetylcholine *in vivo*. Therefore, they hypothesized that the residual bradycardic effect of adenosine and acetylcholine in the knockout mice may be due to decreases in slow depolarizing currents, namely cationic (I_f_), and sustained inward (I_*ST*_) currents, which are directly or indirectly modulated by the cyclic AMP pathway. The opposite was however detected in our hands, where blockade of GIRK/K_IR_3.1/3.4 currents with tertiapin Q reduced, instead of increasing, the whole-cell inward current caused by the A_1_ receptor agonist, R-PIA, in rat atrial cardiomyocytes (Figure 6). Besides ivabradine analogs (e.g., zatebradine), which produce use-dependent inhibition of hyperpolarization-activated mixed Na^+^-K^+^ inward current I_f_ (cyclic nucleotide-gated HCN channel) in sinoatrial node cells but also significantly reduce voltage-gated outward K^+^ currents (IK) at the same concentrations, there are no other specific pharmacological manipulators to dissect the influence of these currents in the spontaneously beating rat atria. Yet, we found no differences in spontaneous atrial rate and inotropy upon applying the cyclic AMP-specific phosphodiesterase type 4 inhibitor rolipram (1–100 μM) (unpublished observations), showing that under the present experimental conditions cyclic nucleotide-gated currents play a minor role. Moreover, *in vivo* data supports a greater role for I_f_ currents in His-Purkinje fibers vs. SAN tissue. In support of our hypothesis, immunolocalization studies with an antibody specific for GIRK1/K_IR_3.1 demonstrates that the distribution of this channel subtype follows a similar pattern of both A_1_ and M_2_ receptors in SA node and atrial cardiomyocytes (Figure 5).

Blockade of voltage-dependent K_v_ with 4-AP and ATP-sensitive K_ATP_/K_IR_6 channels with glibenclamide failed to modify atrial depression caused by adenosine A_1_ and muscarinic M_2_ receptor agonists in spontaneously beating rat atria, when the K^+^ channel inhibitors were applied in concentrations as high as 10 μM. Likewise, 4-AP and blockade of the rapid delayed rectifier potassium current (I_*Kr*_) operated by hERG channels with E4031 did not modify cardiac responses to adenosine in isolated blood-perfused atria of the dog (Oguchi et al., 1995). These findings rule out the involvement of voltage-dependent K_v_ cannels on the inhibitory effects of A_1_ and M_2_ agonists and confirm data from interaction studies between 4-AP (0.3–3 mM) and R-PIA in the guinea-pig using spontaneously beating and electrically-driven atria (De Biasi et al., 1989). Concerning the lack of effect of glibenclamide in antagonizing the responses to M_2_ and A_1_ receptor agonists in rat atria, our results agree with previous reports in the guinea-pig indicating that cardiodepression by these agents are not operated by ATP-sensitive K_ATP_/K_IR_6 channels (Urquhart et al., 1993; Ford and Broadley, 1999).

Due to high sensitivity of K_Ca_2/SK channels for Ca^2+^ activation yielding half maximal activation at ~300 nM [Ca^2+^]_*i*_ with a Hill coefficient between 4 and 5 (Xia et al., 1998), these channels aid in integrating changes in intracellular free Ca^2+^ concentration with membrane potential in the late phase of cardiac repolarization. The small conductance Ca^2+^-activated SK potassium channel subfamily differs in apamin sensitivity, with the SK2 (K_Ca_2.2) being more sensitive (IC_50_ ~ 70 pM) than the SK3 (K_Ca_2.3) (IC_50_ ~ 0.63–6 nM), while a more pronounced expression of these channels exist in atria compared with ventricle myocytes of different species (Xu et al., 2003; Jager and Grissmer, 2004; Tuteja et al., 2005). Data from immunolocalization studies indicate that the apamin-sensitive SK2 (K_Ca_2.2) channel seems to be more abundant in atrial cardiomyocytes than in the SA node (Figure 5), while the opposite is observed with the SK3 (K_Ca_2.3) channel (cf. Tuteja et al., 2005). Preferential co-localization of SK2 (K_Ca_2.2) channels and A_1_ receptors in atrial cardiomyocytes, rather than in SA node, might explain why apamin sensitized (by more than 10 fold) atria to the negative inotropic action of the A_1_ receptor agonist, R-PIA, without significantly affecting the chronotropic effect of the nucleoside. In theory, the leftward shift caused by apamin of the concentration-response curve of R-PIA regarding its negative inotropic effect was unexpected, considering that inhibition of repolarizing K_Ca_2/SK currents should prolong the action potential duration and, thereby, increase the time available for Ca^2+^ influx through Ca_v_1 (L-type) channels leading to a positive inotropic response. In fact, apamin alone caused a mild (< 20%) positive inotropic response (with no effect on chronotropy) which was counteracted by the Ca_v_1 (L-type) channel inhibitor, verapamil (Figure 11). Controversy still exists in the literature regarding the effect of K_Ca_2/SK channel blockers on action potential duration that derive from interspecies differences, experimental idiosyncrasies (e.g., paced vs. unpaced, high vs. low pacing rates), uneven expression of channels throughout atria and unusual pharmacological profiles due to SK2-SK3 channels heteromerization (see e.g., Xu et al., 2003; Nagy et al., 2009; Hancock et al., 2015). Apart from this dispute, we agree with other authors that action potential repolarization is controlled by a, not yet fully characterized, fine balance between various transmembrane ionic currents that are essential to determine the duration of the cardiac action potential.

One possible explanation for the above disparity could be that blockade of K_Ca_2/SK channels stabilizes the K^+^ gradient across the cell membrane at a higher level (Xu et al., 2003), leading to increased GIRK/K_IR_3.1/3.4-mediated K^+^ efflux and cell hyperpolarization *per* G protein-coupled receptor activated, thus affecting the relative potency of the A_1_ receptor agonist. If this were the case, apamin should have also potentiated the negative inotropic effect of the M_2_ receptor agonist in a similar manner to that observed with the A_1_ agonist, taking into consideration that both receptors couple to GIRK/K_IR_3.1/3.4 channels (Kurachi et al., 1986). However, atrial depression caused by oxotremorine was not affected by apamin, indicating that Ca^2+^-activated K_Ca_2/SK channels are not involved in muscarinic M_2_ receptors activity. Activation of K_Ca_2/SK currents causes a hyperpolarizing leak of potassium ions from the cell in favor of its concentration gradient. Therefore, increases in the extracellular potassium concentration should mimic blockage of these currents by apamin. Coincidently, both apamin and augmentation of the extracellular concentration of potassium (from 2.7 to 4.7 mM) sensitized atria to the negative inotropic effect of the A_1_ receptor agonist, without affecting adenosine negative chronotropy. Neither apamin nor increases in the extracellular K^+^ concentration affected significantly oxotremorine-induced cardiodepression in spontaneously beating rat atria. We are aware that increases in extracellular K^+^ has many effects on excitable cells, yet under our experimental conditions this approach positively discriminated the A_1_ receptor-mediated negative inotropic effect by shifting to the left the concentration-response curve of the adenosine analog, R-PIA, without affecting the muscarinic atrial depression.

The most plausible explanation might be that inactivation of K_Ca_2/SK outward currents caused by adenosine A_1_ receptors (via G protein α subunit) in the late phase of atrial repolarization may prolong the action potential duration and shift the resting membrane potential of cardiomyocytes toward more depolarized potentials resulting in an increase in the net Ca^2+^ influx through Ca_v_1 (L-type) channels (cf. Lu et al., 2007; Skibsbye et al., 2015). This hypothesis may be challenged by a recent report showing that in right atria paced at 5 Hz frequency both acetylcholine and the A_1_ receptor agonist, CPA, shortened action potential duration at 90% repolarization (APD_90_) and the ERP in a tertiapin Q-sensitive manner (Wang et al., 2013). However, in contrast to our work using spontaneously beating atria, these authors found a basal GIRK/K_IR_3 current that was active without exogenous receptor activation, which may be a confounder of results interpretation in the two studies. Moreover, application of tertiapin Q after receptor activation caused APD_90_ and ERP return close to baseline values but it did not gave an overshoot that would be expected if the activation by adenosine and acetylcholine only affected GIRK/K_IR_3-mediated currents. This phenomenon was interpreted as a reflection of multiple downstream targets of G_*i*_ receptors, which may include activation of Ca_v_1.3 (L-type) channels that would contribute to sustain ionotropy (Wang et al., 2013). The molecular mechanisms underlying the coupling of Ca_v_1 and K_Ca_2/SK channels are unknown. Functional coupling of Ca_v_1.3 (L-type) and K_Ca_2/SK channels has been described in atrial myocytes via the cytoskeletal protein α-actinin2, an F-actin cross-linking protein directly bridging C-terminal regions of both channels (Lu et al., 2007). An indirect pathway requiring binding of Ca^2+^ to calmodulin (CaM), which then bounds to a CaM-binding domain on the intracellular subunit of the SK channel, has also been proposed. Here, we showed that adenosine A_1_ receptors exerts a dual role by activating GIRK/K_IR_3 and inactivating K_Ca_ 2/SK mediated outward currents, with the latter being revealed at a test potential of +40 mV after transient activation of high-voltage Ca_v_1 (L-type) channels by a brief (50 ms) depolarizing pulse to −10 mV (Grant, 2009). Upon blocking GIRK/K_IR_3.1/3.4 channels with tertiapin Q, the A_1_ receptor agonist decreased, rather than increased, the outward component and this effect was fully prevented by co-application of the K_Ca_2/SK channel blocker apamin. These findings clearly indicate that the net GIRK/K_IR_3.1/K_IR_3.4 outward current triggered by adenosine that is responsible for reducing the SA node automatism may be partially counteracted by the inactivation of an apamin-sensitive Ca^2+^-activated K_Ca_2/SK repolarizing current leading to prolongation of action potential duration and to Ca^2+^ influx into atrial cardiomyocytes via voltage-gated Ca_v_1 (L-type) channels. On its own, Ca^2+^ influx into cardiomyocytes may also influence action potential duration through Ca^2+^-sensitive ionic currents, such as the sodium-calcium exchanger and the calcium-sensitive chloride current. Although there are several studies addressing the effects of adenosine on potassium currents in atrial myocytes (Kurachi et al., 1986; Banach et al., 1993; Wellner-Kienitz et al., 2000; Lomax et al., 2003), to the best of our knowledge there is no report showing that activation of the adenosine A_1_ receptor leads to inactivation of an outward K^+^ current carried out by K_Ca_2/SK channels in these cells.

The (patho)physiological role of apparent opposite effects on potassium currents caused by adenosine A_1_ receptors activation is under debate. Our study suggests that inhibition of K_Ca_2/SK channels by adenosine plays a remarkable role on atrial inotropy. These findings seem to be reliable because blockade of K_Ca_2/SK channels cause a delay in the late phase of the cardiac repolarization (Xu et al., 2003; Tuteja et al., 2005; but see Nagy et al., 2009; Hancock et al., 2015). According to the most accepted hypothesis to explain negative inotropic effects upon GIRK/K_IR_3.1/3.4 channels (Wang and Belardinelli, 1994; Ford and Broadley, 1999), inhibition of cardiac repolarizing K^+^ currents, such as the apamin-sensitive small conductance Ca^2+^-activated K_Ca_2/SK current, may increase the time available for Ca^2+^ influx via Ca_v_1 (L-type) channels due to action potential prolongation and this might counteract the negative inotropic effects promoted by K^+^ efflux through GIRK/K_IR_3.1/3.4 channels. In addition to the enrolment of K_Ca_2/SK channels on atrial inotropic mechanisms, these channels have been identified as key players in the course of supraventricular arrhythmias (Li et al., 2009; Yu et al., 2012), which is particularly interesting in a context of anti- and pro-arrhythmic properties of adenosine and its derivates (Kabell et al., 1994; Bertolet et al., 1997; Lim et al., 2009). Further studies remain to be performed to elucidate if K_Ca_2/SK channels are exclusively modulated by adenosine A_1_ receptors, but at this moment our data suggests that cardiac muscarinic M_2_ receptors do not play a role in this mechanism.

Besides the involvement of various K^+^ channel subtypes, the electrophysiological activity of atrial myocytes involves Ca^2+^ influx by Ca_v_1 (L-type) voltage-sensitive channels (Amin et al., 2010). Novel studies describe the Ca_v_1.3 channel as a determinant of human heart rate but not of ventricular excitation-contraction coupling, which depends mainly on Ca^2+^ influx through the Ca_v_1.2 channel isoform (Baig et al., 2011). Confocal microscopy data showed that immunoreactivity against Ca_v_1 (L-type), using an antibody specific for the regulatory α_2_δ subunit of the channel that does not target subtype-specific α_1_ subunits, was consistently distributed in both SA node and right atrium. Voltage-sensitive Ca_v_1 (L-type) channels are blocked by organic antagonists. These include dihydropyridines (nifedipine) and phenylalkylamines (verapamil), but only the second has cardioselective activity with useful clinical indications to normalize tachycardia rhythms (Grant, 2009). However, little is known about the functional interactions between adenosine and Ca_v_1 (L-type) channel blockers on atrial activity despite the two compounds are often used in line to revert supraventricular dysrhythmias. Our findings clearly demonstrate for the first time that blockade of Ca_v_1 (L-type) channels by nifedipine and verapamil predisposes atria to the negative inotropic action of adenosine probably by uncoupling A_1_-receptor-mediated K_Ca_2/SK channel inhibition from its effector system, the Ca_v_1 (L-type) subtype channel. Further studies are required to investigate the interplay of the two channels *vis a vis* A_1_ receptors activation. Contrariwise, nifedipine and verapamil were devoid of effect on muscarinic M_2_-receptor-mediated actions on spontaneously beating rat atria. This disparity is in agreement with literature. Unlike the A_1_ receptor, activation of the muscarinic M_2_ receptor in the heart might not affect Ca_v_1 (L-type) currents (De Biasi et al., 1989; Song and Belardinelli, 1994; Belardinelli et al., 1995), although both receptors may co-localize and share a common pathway leading to hyperpolarization of atrial myocytes via K^+^ efflux through GIRK/K_IR_3.1/3.4 channels (Belardinelli and Isenberg, 1983; Kurachi et al., 1986).

Receptor reserve refers to a phenomenon whereby stimulation of only a fraction of the whole receptor population apparently elicits the maximal effect achievable in a particular tissue depending on agonists efficacy and on the pathways activated to cause signal amplification (reviewed in Dhalla et al., 2003; Kenakin, 2009). If the receptor reserve is small for a given agonist, the agonist will only elicit the effect in a significant extent when used at high concentrations, while the same agonist can produce the effect even in low concentrations if the receptor reserve is high. Previous studies indicate that atrial cardiomyocytes possess a substantial A_1_ receptor reserve for the direct negative inotropy, which is greater than the A_1_ receptor reserve for any other effects in the guinea-pig atrium paced electrically at 3 Hz-frequency (Gesztelyi et al., 2013; Kiss et al., 2013). Taking this into account, one would predict that enzymatically-stable full A_1_ receptor agonists, such as R-PIA, should be more potent in decreasing the contractile force than the atrial rate. Yet, we show here that bradycardia produced by R-PIA predominates over the negative inotropic effect of the A_1_ receptor agonist in spontaneously beating isolated rat atria. Whether increases in atrial A_1_ receptor reserve specifically for the direct negative inotropy occur in the presence of the K_Ca_2/SK blocker, apamin, or of Ca_v_1 (L-type) channel inhibitors, nifedipine or verapamil, deserve future investigations as they may sensitize atria to mechanical depression in clinical conditions requiring administration of adenosine followed by verapamil for conversion of paroxysmal supraventricular tachycardia.

## Conclusion

In conclusion, this study contributes to elucidate the pharmacology of the ionic mechanisms responsible for adenosine chronoselectivity via adenosine A_1_ receptors activation in spontaneously beating rat atria as compared with other cardiodepressant agents, like those activating muscarinic M_2_ receptors and inhibiting voltage-sensitive Ca_v_1 (L-type) channels. Activation of the adenosine A_1_ receptor decreases SA node automatism mainly by promoting K^+^ efflux through βγ subunits of G protein-coupled inwardly rectifying GIRK/K_IR_3 channels. This effect, may be counteracted by inhibition of K_Ca_2/SK currents (probably via G protein α subunit) leading to a subsequent prolongation of atrial repolarization. The increase in the time available for Ca^2+^ influx through voltage-sensitive Ca_v_1 (L-type) channels may be essential to sustain inotropy in the presence of the adenosine A_1_ receptor agonist in concentrations causing significant negative chronotropic actions. We are aware that this hypothesis is not consensual therefore detailed electrophysiological studies are warranted to determine precisely whether or not activation of the adenosine A_1_ receptor contributes to prolongation of action potentials in atrial cardiomyocytes. Although Ca^2+^ transients are certainly important determinants of atrial contractility, further studies are required to investigate how they integrate with mechanisms regulating the ultimate step of cardiac contractility, the myofilament Ca^2+^ sensitivity and cross-bridging, which may also conceivably be altered by A_1_ receptors stimulation (see e.g., Strang et al., 1995). Even so, our findings might be of clinical relevance, as conversion of paroxysmal supraventricular tachycardia to sinus rhythm may involve different sequences of intravenous drugs administrations including adenosine (or its stable derivative, tecadenoson) and/or Ca_v_1 (L-type) channel inhibitors (e.g., verapamil) (Lim et al., 2009). Thus, loss of adenosine chronoselectivity and atrial sensitization to the negative inotropic action of A_1_ receptors by Ca_v_1 (L-type) channel blockers may have deleterious effects in critical patients whenever adenosine is used concomitantly with verapamil.

## Author contributions

BB, NO, FF, PL, MF, AF, and PC contributed significantly for the experimental design, data acquisition, and interpretation of the results obtained; BB, NO, FF, PL, MF, AF, and PC drafted and revised the manuscript; all authors approved the submitted version of the manuscript and agreed that all aspects of the work are accurate. BB and NO equally contributed to this work and should be named first co-authors.

## Funding

This work was partially funded by the Foundation for Science and Technology of Portugal (FCT) within the framework of projects FCOMP-01-0124-FEDER-028726 (FEDER, COMPETE, FCT PTDC/DTP-FTO/0802/2012) and PEst-OE/SAU/UI0215/2014. BB was in receipt of a Young Investigator Fellowship from FCT (BII/UMIB-ICBAS/2009-2).

### Conflict of interest statement

The authors declare that the research was conducted in the absence of any commercial or financial relationships that could be construed as a potential conflict of interest.

## Supplementary material

Supplementary Figure 1 **Concentration-response curves of adenosine on rate (chronotropy) and mechanical tension (inotropy) of spontaneously beating atria**. Adenosine (0.001–3 mM) was applied once every 2 min at increasing concentrations. The ordinates are percentage of variation from baseline spontaneous contractions. The data are expressed as mean ± SEM from an *n* number of individual experiments. ^*^*P* < 0.05 compared with adenosine-induced percentage of baseline variation on chronotropy.

Supplementary Figure 2 **Effect of increasing the extracellular concentration of potassium from 2.7 (Control) to 4.7 mM on the negative chronotropic and inotropic effects of oxotremorine (A,B) and R-PIA (C,D) on spontaneously beating rat atria**. The ordinates are percentage of variation of spontaneous contraction rate (chronotropic effect, **A,C**) and mechanical tension (inotropic effect, **B,D**) compared to baseline values obtained before increasing the KCl concentration to 4.7 mM. Data are expressed as mean ± SEM from an *n* number of individual experiments. ^*^*P* < 0.05 compared with the effect of oxotremorine or R-PIA in control conditions.

Supplementary Figure 3 **Higher-magnification representative confocal micrographs of rat right atrium and SA node sections shown in Figure 5**. Immunofluorescence stainings for K_IR_3.1 (GIRK1) and Ca_V_α_2_δ−1 channel subunits appear in green. Cell nuclei are stained in blue with DAPI. The corresponding differential interference contrast (DIC) images are also shown for comparison (last row). Similar results were obtained in five additional experiments.

Supplementary Figure 4 **Higher-magnification representative confocal micrographs of rat right atrium and SA node sections shown in Figure 5**. Immunofluorescence stainings for K_Ca_2.2 (SK2) and K_Ca_2.3 (SK3) channel subunits appear in green. Cell nuclei are stained in blue with DAPI. The corresponding differential interference contrast (DIC) images are also shown for comparison (last row). Similar results were obtained in five additional experiments.
