# Supplementary figures and images for: Ion Fluxes through KCa2 (SK) and Cav1 (L-type) Channels Contribute to Chronoselectivity of Adenosine A1 Receptor-Mediated Actions in Spontaneously Beating Rat Atria
Source: Front Pharmacol. 2016 Mar 7;7:45. doi: 10.3389/fphar.2016.00045 (PMC4780064; doi:10.3389/fphar.2016.00045)

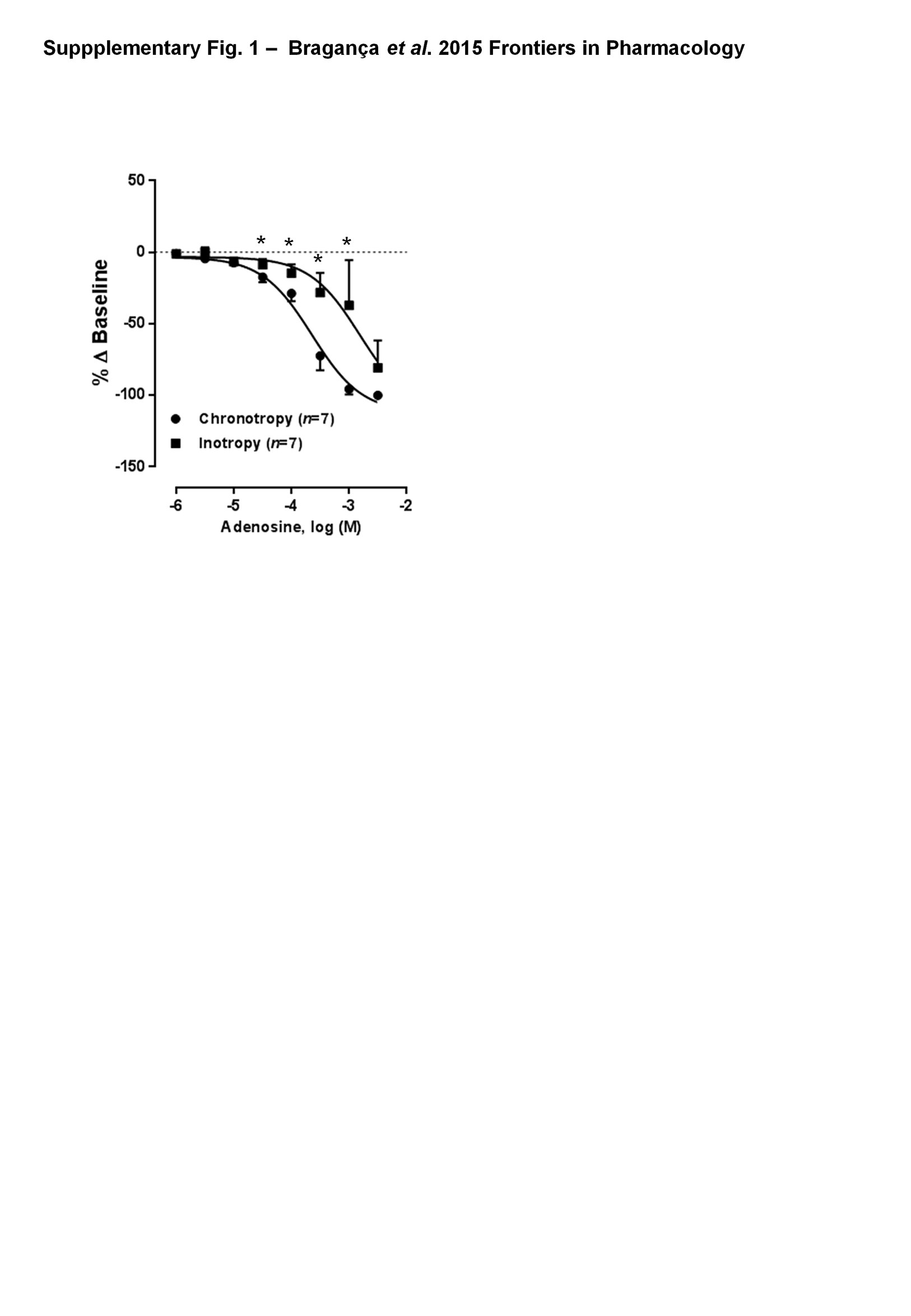

Supplement: Supplementary Figure 1 — Concentration-response curves of adenosine on rate (chronotropy) and mechanical tension (inotropy) of spontaneously beating atria. Adenosine (0.001–3 mM) was applied once every 2 min at increasing concentrations. The ordinates are percentage of variation from baseline spontaneous contractions. The data are expressed as mean ± SEM from an n number of individual experiments. *P < 0.05 compared with adenosine-induced percentage of baseline variation on chronotropy. [file Image1.tif]

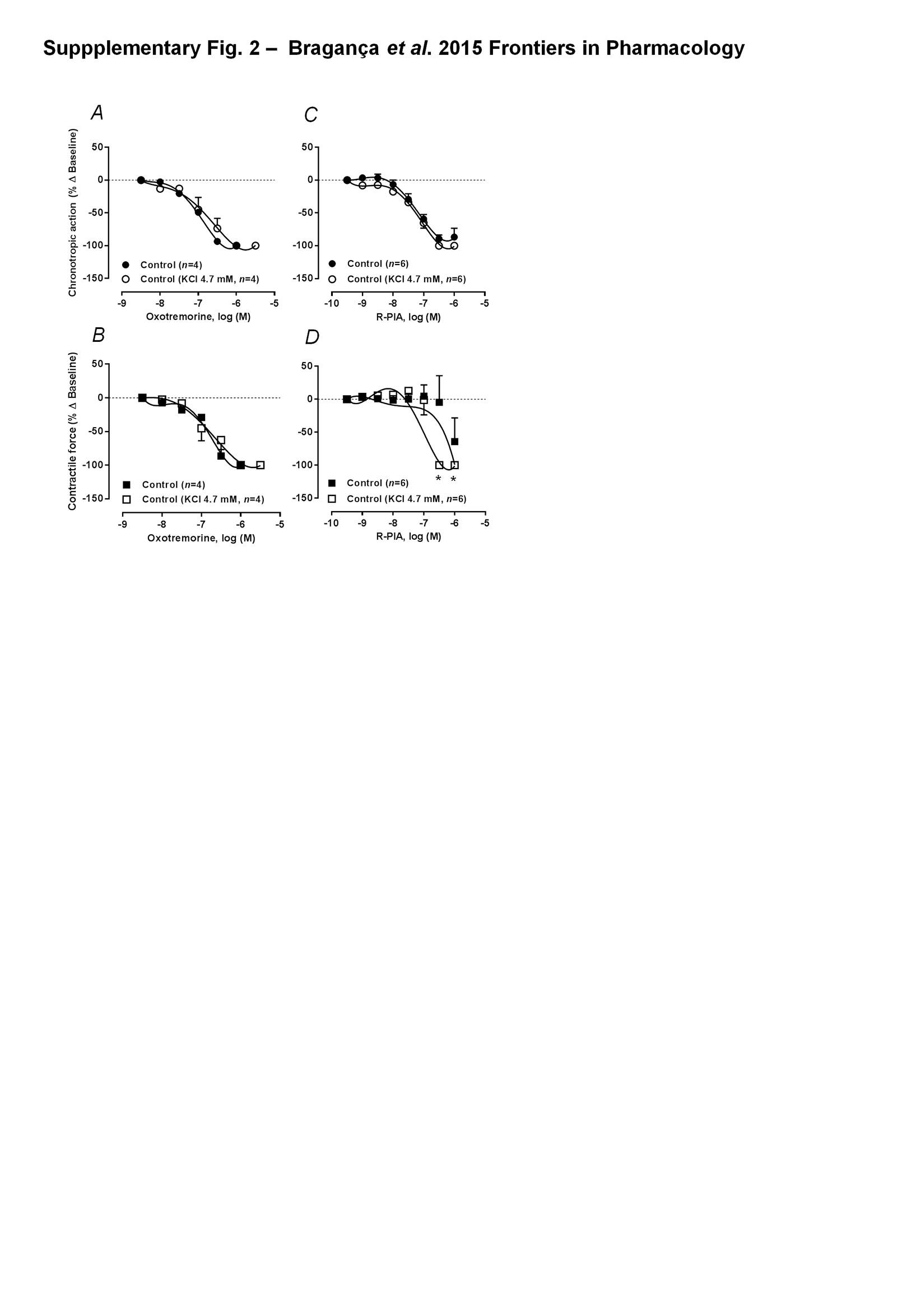

Supplement: Supplementary Figure 2 — Effect of increasing the extracellular concentration of potassium from 2.7 (Control) to 4.7 mM on the negative chronotropic and inotropic effects of oxotremorine (A,B) and R-PIA (C,D) on spontaneously beating rat atria. The ordinates are percentage of variation of spontaneous contraction rate (chronotropic effect, A,C) and mechanical tension (inotropic effect, B,D) compared to baseline values obtained before increasing the KCl concentration to 4.7 mM. Data are expressed as mean ± SEM from an n number of individual experiments. *P < 0.05 compared with the effect of oxotremorine or R-PIA in control conditions. [file Image2.tif]

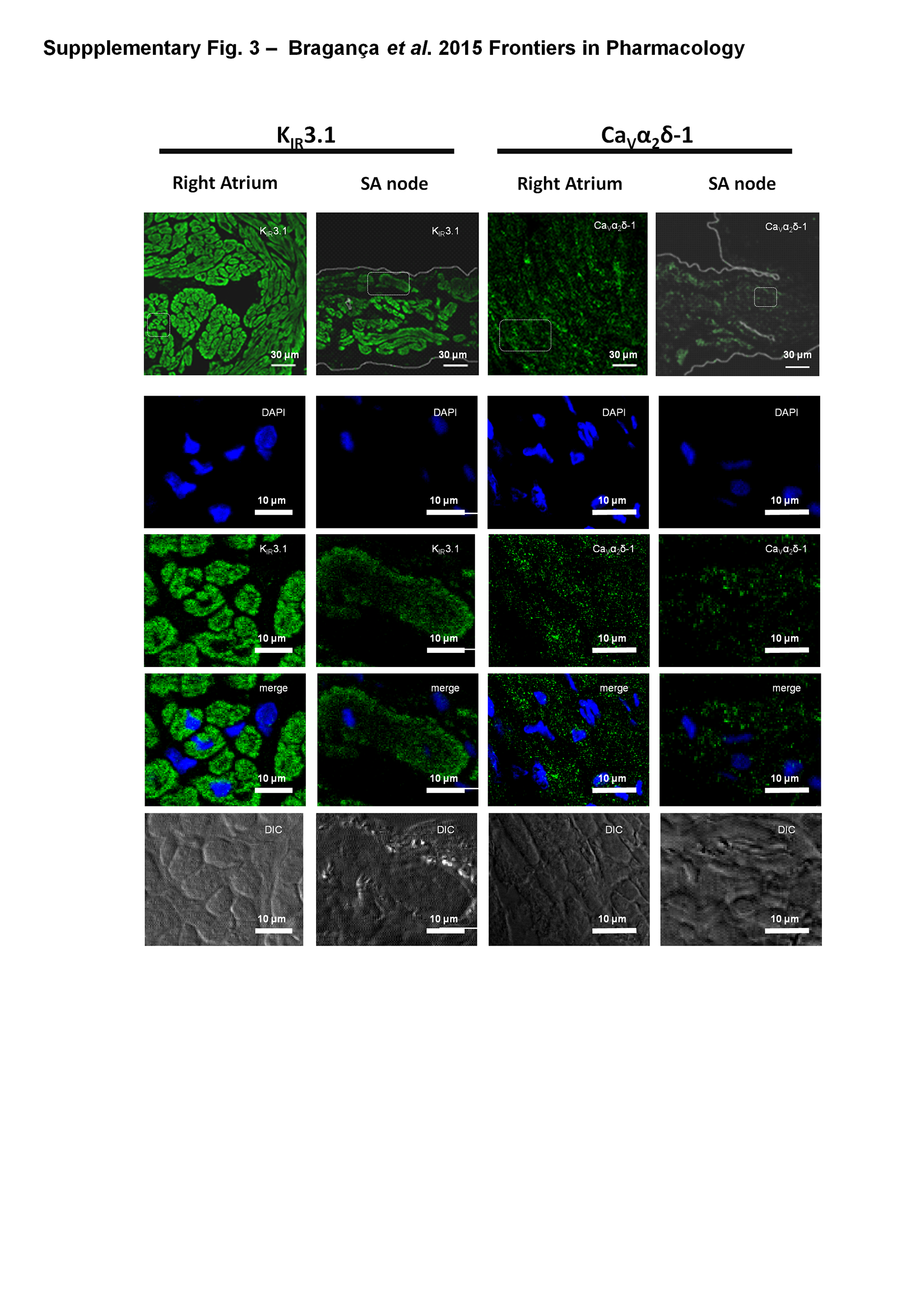

Supplement: Supplementary Figure 3 — Higher-magnification representative confocal micrographs of rat right atrium and SA node sections shown in Figure 5. Immunofluorescence stainings for KIR3.1 (GIRK1) and CaVα2δ−1 channel subunits appear in green. Cell nuclei are stained in blue with DAPI. The corresponding differential interference contrast (DIC) images are also shown for comparison (last row). Similar results were obtained in five additional experiments. [file Image3.tif]

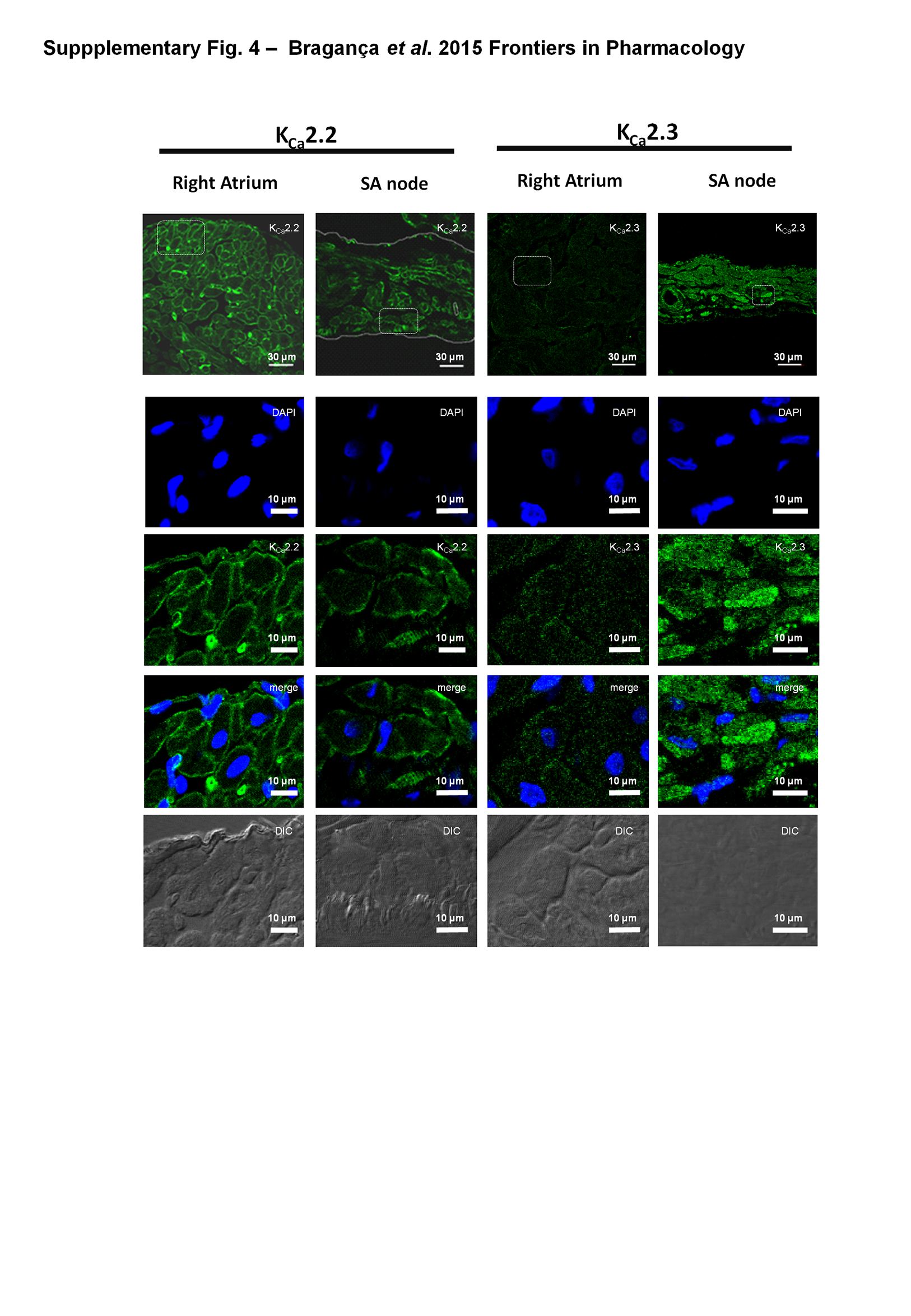

Supplement: Supplementary Figure 4 — Higher-magnification representative confocal micrographs of rat right atrium and SA node sections shown in Figure 5. Immunofluorescence stainings for KCa2.2 (SK2) and KCa2.3 (SK3) channel subunits appear in green. Cell nuclei are stained in blue with DAPI. The corresponding differential interference contrast (DIC) images are also shown for comparison (last row). Similar results were obtained in five additional experiments. [file Image4.tif]
